# Tuning the Properties of Protein-Based Polymers Using High-Performance Orthogonal Translation Systems for the Incorporation of Aromatic Non-Canonical Amino Acids

**DOI:** 10.3389/fbioe.2022.913057

**Published:** 2022-05-30

**Authors:** Osher Gueta, Ortal Sheinenzon, Rotem Azulay, Hadas Shalit, Daniela S. Strugach, Dagan Hadar, Sigal Gelkop, Anat Milo, Miriam Amiram

**Affiliations:** ^1^ Avram and Stella Goldstein-Goren Department of Biotechnology Engineering, Ben-Gurion University of the Negev, Beersheba, Israel; ^2^ Department of Chemistry, Ben-Gurion University of the Negev, Beersheba, Israel

**Keywords:** non-canonical amino acids (ncAAs), elastin-like polypeptides (ELP), resilin-like polypeptides (RLP), smart biomaterials, genetic code expansion

## Abstract

The incorporation of non-canonical amino acids (ncAAs) using engineered aminoacyl-tRNA synthetases (aaRSs) has emerged as a powerful methodology to expand the chemical repertoire of proteins. However, the low efficiencies of typical aaRS variants limit the incorporation of ncAAs to only one or a few sites within a protein chain, hindering the design of protein-based polymers (PBPs) in which multi-site ncAA incorporation can be used to impart new properties and functions. Here, we determined the substrate specificities of 11 recently developed high-performance aaRS variants and identified those that enable an efficient multi-site incorporation of 15 different aromatic ncAAs. We used these aaRS variants to produce libraries of two temperature-responsive PBPs—elastin- and resilin-like polypeptides (ELPs and RLPs, respectively)—that bear multiple instances of each ncAA. We show that incorporating such aromatic ncAAs into the protein structure of ELPs and RLPs can affect their temperature responsiveness, secondary structure, and self-assembly propensity, yielding new and diverse families of ELPs and RLPs, each from a single DNA template. Finally, using a molecular model, we demonstrate that the temperature-responsive behavior of RLPs is strongly affected by both the hydrophobicity and the size of the unnatural aromatic side-chain. The ability to efficiently incorporate multiple instances of diverse ncAAs alongside the 20 natural amino acids can help to elucidate the effect of ncAA incorporation on these and many other PBPs, with the aim of designing additional precise and chemically diverse polymers with new or improved properties.

## Introduction

Incorporating non-canonical amino acids (ncAAs) into proteins has emerged as a powerful methodology to improve, alter, or introduce new functions into proteins. The incorporation of ncAAs bearing a variety of chemical groups can facilitate the elucidation of protein structure–function relationship (Debelouchina and Muir, 2017; Chen et al., 2018) or protein–protein interactions (Nguyen et al., 2018), or the production of protein-based therapeutics (Huang and Liu, 2018) and biomaterials with novel functions (Connor and Tirrell, 2007; Israeli et al., 2019), among many other applications (Johnson et al., 2010; Chin, 2017; Voller and Budisa, 2017; Young and Schultz, 2018; Lee et al., 2019; Zhou and Deiters, 2021). There are two common strategies for expressing recombinant proteins containing ncAAs. The first entails substituting one of the natural AAs with an ncAA. This technique permits the incorporation of multiple instances of a ncAA into the target protein and in entire proteomes (Johnson et al., 2010). However, substitution allows the incorporation of only 19 natural AAs alongside the ncAA, and the ncAA must be a close analog of the natural AA that it replaces, therefore using this approach limits the flexibility of protein engineering. Alternatively, ncAAs can be incorporated by codon reassignment or frameshift codons using orthogonal translation systems consisting of an aminoacyl-tRNA synthetase (aaRS) and a tRNA pair that do not interact with the native aaRSs and tRNAs in the expression host. This technique has enabled the incorporation of >200 different ncAAs, using aaRS·tRNA pairs such as the tRNA^Tyr^·TyrRS pair from *Methanocaldococcus jannaschii* (Kobayashi et al., 2003) or the tRNA^Pyl^·PylRS pair from various methanogenic archaea and bacteria, such as from the *Methanosarcina* or *Methanomethylophilus* spp. (Wan et al., 2014; Willis and Chin, 2018). Typically, a TAG stop codon is assigned to the ncAA and the orthogonal tRNA anticodon is mutated to CUA (if needed). In addition, the evolution of the aaRS by mutagenesis of its AA-binding site is required to accommodate the ncAA. However, traditional evolution methodologies typically yield aaRS variants capable of introducing the ncAA at only one or a few instances within a polypeptide chain, and often with reduced yields compared to wild-type proteins (O'Donoghue et al., 2013; Vargas-Rodriguez et al., 2018).

We recently developed an aaRS evolution platform that utilizes multiplex automated genome engineering (MAGE) to create a library of chromosomal MjTyrRS variants. High-performance aaRS variants were selected from these libraries, enabling multi-site incorporation (10–30 instances per protein) of the aromatic ncAAs p-acetyl-phenylalanine (pAcF) (Amiram et al., 2015), p-azido-phenylalanine (pAzF) (Amiram et al., 2015), 4-propargyloxy-L-phenylalanine (pPR) (Hadar et al., 2021), and phenylalanine-4‘-azobenzene (AzoPhe) (Israeli et al., 2021). Although these aaRS variants were selected based on their ability to incorporate the ncAAs mentioned above, it had been previously demonstrated by us and others that most aaRS variants exhibit some degree of poly-specificity, that is, they can recognize and charge their cognate tRNA with ncAAs bearing various chemical groups (Young et al., 2011; Amiram et al., 2015). The elucidation of the substrate specificities of high-performance aaRS variants can be a facile means for generating highly efficient aaRS–ncAA pairs for the multi-site incorporation of additional ncAAs. Such an expanded set of aaRS–ncAA pairs for the efficient incorporation of multiple instances of ncAAs within a single protein chain can enable the engineering of proteins with the desired new or improved functions and their high-yield production (Ohtake et al., 2015; Rezhdo et al., 2019; Hayashi et al., 2021). In addition, analyzing the relationship between the mutations in the aaRS AA-binding site and the corresponding ncAA binding specificities can inform computational models and facilitate the selection of smaller, focused libraries for future aaRS evolution experiments (Hauf et al., 2017; Baumann et al., 2019).

Multi-site ncAA incorporation is particularly desirable for the design and production of protein-based polymers (PBPs), which consist of tandem repeats of either natural or artificial short peptide motifs, and whose properties are determined by the sequence of the AAs and ncAAs in these motifs (Connor and Tirrell, 2007; Israeli et al., 2019; Varanko et al., 2020; Chang et al., 2021). Although the incorporation of ncAAs in numerous PBPs can be used to expand their range of properties and functions (Connor and Tirrell, 2007; Israeli et al., 2019), we focus here on the effect of ncAA incorporation on the properties of two families of bio-inspired, thermo-responsive PBPs: elastin-like polypeptides (ELPs) and resilin-like polypeptides (RLPs). ELPs are arguably the most well-studied family of artificial PBPs that comprise multiple repeats (typically 5–200 repeats) of the VPGXG motif (variations of the tropoelastin-derived VPGVP motif), where X is permissive to any amino acid (AA) except proline (MacEwan and Chilkoti, 2010). ELPs undergo a reversible soluble-to-insoluble phase transition at their lower critical solution temperature (LCST), often termed the transition temperature, which depends on the ELP composition and is maintained in ELP fusion proteins and conjugates (MacEwan and Chilkoti, 2010; Amiram et al., 2013; MacEwan and Chilkoti, 2014; Despanie et al., 2016). Extensive characterization of the mechanism underlying this phase transition permits the prediction and tuning of the LCST of the ELP, primarily by varying the hydrophobicity of the X-guest residue and the molecular weight (MW) of the ELP (Meyer and Chilkoti, 2004; McDaniel et al., 2013). RLPs are artificial, repetitive PBPs, comprising sequences identical to, or inspired by, motifs found in natural resilin proteins (Balu et al., 2021). RLPs exhibit a temperature-responsive behavior characterized by an upper critical solution temperature (UCST), with some variants also showing an additional LCST-type phase transition. The role of aromatic AAs in the UCST- and LCST-type transition behaviors appears to have particular significance (Martin and Mittag, 2018). The LCST of ELPs is affected mainly by hydrophobic interactions (Pak et al., 2016; Ruff et al., 2018) but may also be affected by hydrogen bonding and π-π stacking of the aromatic side-chains (Taylor et al., 2020). In contrast, several studies have demonstrated that the UCST transition appears to be strongly influenced by the fraction, position, and identity of aromatic AAs and their interactions with other AAs (e.g., cation-π interactions), as well as by the overall hydrophobicity (Joseph et al., 2021).

In this work, we have sought to characterize the substrate specificities of 11 of our previously described, MAGE-evolved aaRS variants using 38 different aromatic ncAAs. We identified efficient aaRS variants that are capable of incorporating 15 of these ncAAs in up to 30 instances in a single ELP chain. We applied this information to produce ELP and RLP libraries bearing multiple instances of each ncAA and characterized the effect of the newly introduced chemical group on the properties of these PBPs.

## Materials and Methods

### Materials

ncAAs were purchased from various vendors, as indicated in Supplementary Table S1. Plasmid purification was conducted with Plasmid HiYield mini-prep (RBC Bioscience). SDS solution was purchased from Bio-Rad. Anhydrotetracycline hydrochloride was purchased from Sigma-Aldrich. Arabinose and 2xYT media were purchased from Tivan Biotech, and IPTG from Biolab-Biology. C321. ΔA (Isaacs lab) was a gift from Farren Isaacs (Addgene plasmids # 73,581). Isomerization experiments were performed with a 365 nm UV lamp (VL-6.LC, 12W, Vilber Lourmat) and blue mounted LEDs: 405 nm, 1,000 mW, 800 mA (M405L4, Thor-Labs). Plasmids encoding the pAcFRS.1.t1, pAcFRS.2.t1, pAzFRS.1.t1, pAzFRS.2.t1, Mut1-RS, Mut2-RS, and AzoRS-4 can be obtained from Addgene.

### Analysis of GFP Expression by Intact-Cell Fluorescence Measurements

For 96-well plate-based assays, strains harboring plasmids encoding the orthogonal translation systems and ELP-GFP reporter plasmids were inoculated with transformed cells from either a fresh agar plate or from stocks stored at −80°C, and grown to confluence overnight. Cultures were then inoculated at a 1:30 dilution in 2xYT medium supplemented with kanamycin (30 μg ml^−1^), chloramphenicol (25 μg ml^−1^), and the ncAA (0.25 mM of ncAAs 1–5 or 1 mM of all other ncAAs). The expression of the aaRS was induced by adding arabinose (0.2%). Cells were allowed to grow at 34°C to an OD_600_ of 0.5–0.8 in a shaking plate incubator at 550 rpm (∼5 h) and GFP expression was induced by adding anhydrotetracycline (60 ng ml^−1^). Following overnight expression, the cells were centrifuged at 4,000 rpm for 3 min, the supernatant medium was removed, and the cells were resuspended in PBS. GFP fluorescence was measured on a Biotek spectrophotometric plate reader by using excitation and emission wavelengths of 485 nm and 528 nm, respectively. Fluorescence signals were normalized by dividing the fluorescence counts by the OD_600_ reading. Shown are representative results of at least three different assays conducted for each reporter protein and expression condition.

### ELP Expression and Purification

Before batch expression, starter cultures (1:40 v/v of final expression volume) of 2xYT media, supplemented with kanamycin (30 μg ml^−1^) and chloramphenicol (25 μg ml^−1^), were inoculated with transformed cells from either a fresh agar plate or from stocks stored at −80°C, incubated overnight at 34°C while shaking at 220 rpm, and transferred to expression flasks containing 2xYT media, antibiotics, arabinose (0.2%), and the ncAA (0.25 mM of ncAAs 5 or 1 mM of all other ncAAs). Cells were allowed to grow to an OD_600_ of 0.5–0.8 and protein expression was induced with isopropyl β-d-1-thiogalactopyranoside (IPTG, 1 mM). The cells were harvested 24 h after inoculation by centrifugation at 4,000 g for 30 min at 4°C. The cell pellet was then resuspended by vortex in milli-Q water (∼4 ml) and either stored at −80°C or purified immediately. For purification, resuspended pellets were lysed by ultrasonic disruption (18 cycles of 10 s sonication, separated by 40 s intervals of rest). Poly (ethyleneimine) was added (0.2 ml of a 10% solution) to each lysed suspension before centrifugation at 4,000 rpm for 15 min at 4°C to separate cell debris from the soluble cell lysate. All ELP constructs were purified by a modified inverse transition cycling (ITC) protocol (Hassouneh et al., 2010) consisting of multiple “hot” and “cold” spins by using sodium chloride to trigger the phase transition. Before purification, the soluble cell lysate was incubated for up to 2 min at 42–65°C to denature the native *E. coli* proteins. The cell lysate was then cooled on ice, centrifuged for 2 min at ∼14,000 rpm, and the pellet was discarded. For “hot” spins, the ELP phase transition was triggered by adding sodium chloride to the cell lysate or to the product of a previous cycle of ITC at a final concentration of up to ∼5 M. The solutions were then centrifuged at ∼14,000 rpm for 10 min and the pellets were resuspended in milli-Q water, after which a 2 min “cold” spin was performed without sodium chloride to remove denatured contaminants. Additional rounds of ITC were conducted as needed using a saturated solution of sodium chloride until sufficient purification was achieved. Purified proteins were visualized on SDS-PAGE.

Protein concentrations were calculated by measuring the OD_280_ of the purified protein according to the extinction coefficients in Supplementary Table S2. Predicted LogD values of the ncAAs were determined using ChemAxon logD predictor (Kubyshkin, 2021).

### RLP Expression and Purification

Batch expression was performed as described above for ELP production. The cells were harvested 24 h after inoculation by centrifugation at 4,000 g for 30 min at 4°C. Purification was performed according to a previously described protocol (Dzuricky et al., 2020). Purified proteins were visualized on SDS-PAGE. Protein concentrations were calculated by measuring the OD_280_ of the purified protein in 8M urea according to the extinction coefficients in Supplementary Table S2.

### Intact Mass Measurements

The intact mass of ELP and RLP variants was measured using MALDI-TOF/TOF autoflex speed at the Ilse Katz Institute for Nanoscale Science and Technology (Ben-Gurion University of the Negev). Spectrum analysis was performed by the Flexanalysis software.

### Phase Transition Analysis

To characterize the inverse transition temperature of the ELP and RLP variants, the OD_600_ of the ELP solution was monitored as a function of temperature, with heating and cooling performed at a rate of 1°C min^−1^ on a UV-vis spectrophotometer equipped with a multicell thermoelectric temperature controller (Thermo Scientific).

### Dynamic Light Scattering (DLS) Analysis

ELP and RLP self-assembly was analyzed using a Zetasizer Nano ZS (Malvern Pananalytical). For each sample, 11–17 acquisitions (determined automatically by the instrument) were obtained at 5°C or 60°C for ELPs or RLPs, respectively. Populations comprising less than 1% of the total mass (by volume) were excluded from the analysis.

### Circular Dichroism (CD) Analysis

The secondary structure of ELPs was studied using an Jasco J-715 spectropolarimeter (Tokyo) equipped with a PTC-348WI temperature controller, using a 1-mm quartz cuvette by scanning from 280 to 180 nm at 5°C. Purified constructs were diluted to 5 μM in water. Data were considered for analysis whenever the Dynode voltage was below 800 V.

## Results and Discussion

### Profiling the Substrate Specificity of High-Performance aaRSs

We first profiled the substrate specificities of our previously selected aaRS variants: pAcFRS.1.t1, pAcFRS.2.t1, and pAzFRS.2.t1 were originally selected for pAcF and pAzF incorporation (Amiram et al., 2015), MutRS-1—MutRS-4 were selected for pPR incorporation (Hadar et al., 2021), and AzoRS-1-AzoRS-4 were selected for AzoPhe incorporation (Israeli et al., 2021). A summary of the mutations in the AA binding site of these aaRS variants, as compared with the parent MjTyrRS, which natively recognizes tyrosine, is provided in Table 1. To identify high-performance aaRSs capable of efficient incorporation of multiple instances of the ncAAs per protein, we assessed the ability of the aaRSs to support the incorporation of either 10 or 30 instances of each ncAA per protein via the expression of two reporter proteins: ELP(10TAG)-GFP and ELP(30TAG)-GFP (Supplementary Table S3). These reporter proteins were produced in the presence of each aaRS·tRNA pair using the *Escherichia coli* strain C321.ΔRF1, which lacks all the native TAG codons and the associated release factors (RF-1) (Lajoie et al., 2013).

**TABLE 1:** Annotations of specific mutations in evolved aaRS variants compared with the WT *Methanocaldococcus jannaschii* tyrosyl-tRNA synthetase (MjTyrRS) sequence. In addition to the indicated mutations, all mutants harbored the R257G and D286R mutations, which have been shown to improve tRNA binding.

Position	32	65	107	108	109	158	159	162	167
aaRS									
Tyr-RS	Y	L	E	F	Q	D	I	L	A
PacFRS.1.t1	L	L	E	F	Q	G	C	R	D
PacFRS.2.t1	L	V	E	F	Q	G	C	R	D
PazFRS.2.t1	L	L	T	Y	M	G	C	R	A
Mut1-RS	L	L	E	F	Q	S	M	K	H
Mut2-RS	L	V	E	F	Q	G	A	E	H
Mut3-RS	T	V	A	Y	M	G	C	R	D
Mut4-RS	L	V	E	F	Q	G	M	S	H
AzoRS-1	G	V	E	F	Q	G	Y	S	F
AzoRS-2	L	V	S	V	S	G	Y	S	F
AzoRS-3	L	V	N	V	L	G	Y	S	F
AzoRS-4	G	V	E	F	Q	G	Y	R	A

Notably, some high-performance aaRS variants, particularly pAcFRS.1.t1 and pAzFRS.2.t1, also exhibit a relatively high incorporation of natural AAs in the absence of their cognate ncAA. This property results in high levels of “background” expression of the ELP-GFP reporters in the absence of a ncAA, although, when present, they accurately incorporate their cognate ncAA (Amiram et al., 2015; Hadar et al., 2021; Israeli et al., 2021). Such background expression can be reduced in conditions that highlight the differences in aaRS efficiencies, such as by increasing the number of TAG codons in the reporter protein, since the aaRSs incorporate the ncAAs more efficiently than the natural AAs. Specifically, relative protein production in the presence of the cognate ncAA, as compared with the absence of any ncAA, is lower for ELP(30TAG)-GFP than for ELP(10TAG)-GFP (Amiram et al., 2015; Hadar et al., 2021). Consequently, we conducted our initial evaluation of multi-site ncAA incorporation using the ELP(30TAG)-GFP reporter to eliminate the possibility of masking the production of ncAA-containing reporter proteins by background expression in the absence of the ncAA.

**FIGURE 1:**
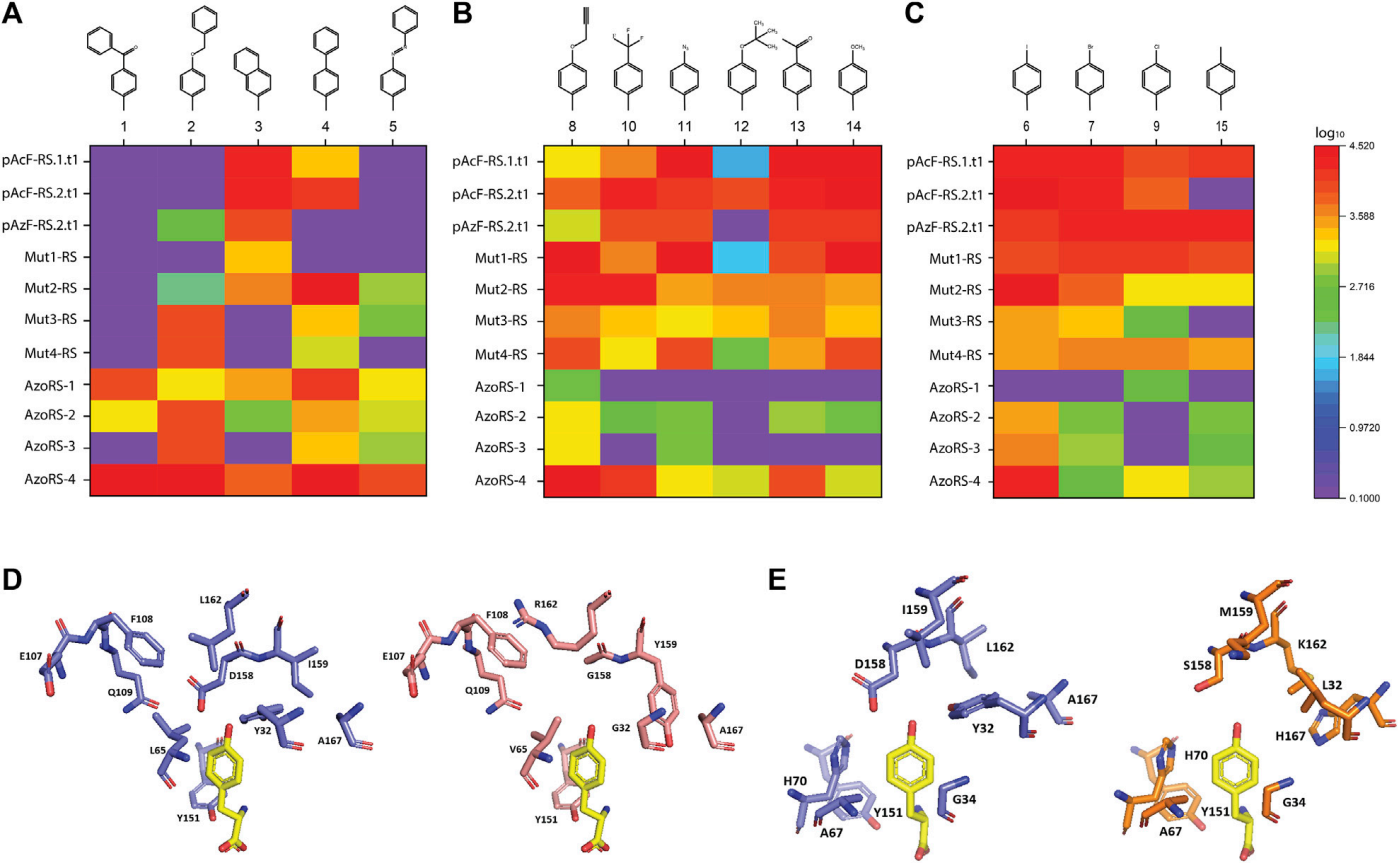
**(A–C)** Specificities of high-performance aaRS variants for the 15 ncAAs depicted in Supplementary Figure S1, assessed by their incorporation into the reporter protein ELP(30TAG)-GFP. **(D–E)** A comparison of the AA-binding pocket, shown in complex with the native cognate AA tyrosine (yellow), of **(D)** native MjTyrRS (purple) compared with AzoRS-4 (pink), and **(E)** native MjTyrRS (purple, different angle) and Mut1-RS (orange). The structural models were generated (based on PDB ID: 1J1U) using the PyMOL program (Schrödinger, LLC).

The expression of the ELP(30TAG)-GFP reporter protein in the presence of 38 different ncAAs revealed that 15 of the ncAAs (including four of the cognate ncAAs for which the aaRS variants were initially selected; Supplementary Figure S1) are efficiently incorporated by one or more of the aaRS variants (Figures 1A–C, Supplementary Figure S2). Notably, all but one (ncAA 3) of the 15 ncAAs that were successfully incorporated are phenylalanine derivatives that harbor 4' (para) substituents. The expression of the ELP(10TAG)-GFP reporter protein in the presence of the same 15 ncAAs confirms the high background incorporation previously observed for some of the variants and reveals that some aaRS variants appear to have increased efficiency in the expression of this reporter, as compared with ELP(30TAG)-GFP (Supplementary Figure S3). This finding is in agreement with previous studies demonstrating that differences in aaRS efficiencies are revealed by increasing the number of TAG codons per protein (Amiram et al., 2015; Hadar et al., 2021; Israeli et al., 2021).

An examination of the specificities of the aaRS variants (indicated as heat maps in Figures 1A–C) reveals several distinctions in the substrate profiles of some of the aaRSs. First, generally, the larger ncAAs (1–5) were suitable substrates for AzoRS-1-4, which were selected for the incorporation of 5 (AzoPhe). In contrast, smaller ncAAs were more efficiently recognized by the remaining aaRSs, which were selected for the incorporation of pAcF (13), pAzF (11), and pPR (8). In addition, while some aaRSs, such as pAcFRS.1.t1, pAcFRS.2.t1, and AzoRS-4, exhibited a broad substrate spectrum and could efficiently accommodate >9 different ncAAs, other aaRSs, such as Mut3-RS, Mut4-RS, AzoRS-1, and AzoRS-3, were more selective and can efficiently aminoacylate only 1-3 ncAAs. We also profiled the substrate specificities of an additional aaRS, namely pAzFRS.1.t1, using our panel of 38 ncAAs. We found that it is exceptionally specific for pAzF and excluded all other ncAAs in our panel (Supplementary Figure S4), in accordance with our previous analysis of this aaRS using another panel of ncAAs (Amiram et al., 2015). Finally, mutually orthogonal aaRS–ncAA pairs, such as AzoRS-1:ncAA1 and Mut2-RS:ncAA6, could be identified from these analyses. Such orthogonal pairs may assist in constructing multiple orthogonal translation systems for the incorporation of two or more ncAAs within a single protein.

A comparison of the mutations in each of the aaRS variants (Table 1) provides information that may aid in the construction of future libraries for aaRS evolution. First, the differences and similarities in the mutations found in the aaRS variants revealed patterns in the types of AAs substituted at certain positions (Figures 1D,E). For example, Y32 and D158, which originally form hydrogen bonds with the tyrosine hydroxyl group, were mutated in all variants to a smaller AA—leucine, threonine, or glycine (for Y32) or glycine and serine (for D158), enlarging the ncAA binding pocket. Similarly, I159 was mutated in all variants to either smaller AAs, such as cysteine or alanine in aaRSs selected for the incorporation of 8, 11, or 13, or to tyrosine in variants selected for the incorporation of 5, where π-π interactions between the side chain of tyrosine 159 and the additional phenyl ring in ncAAs 1–5 may facilitate their binding. Finally, A167 was frequently replaced with a charged AA or with phenylalanine in aaRSs that are compatible with ncAAs bearing the relatively small, or phenyl-based substituents, respectively. Second, although 12 AAs in the AA-binding pocket were targeted for diversification in the selections of all aaRS variants, mutations in only nine of these AAs were evident in the selected aaRSs. It is possible that any changes in the remaining three AAs—G34, A67 and Y151—which remained unaffected in all of our variants, are not beneficial to this group of ncAAs or deleterious to the binding of any AA. A mutagenesis of G34 and A67, which are relatively small AAs, may increase the size of the AA-binding pocket and, thereby, interfere with the binding of aromatic AAs and ncAAs. The mutagenesis of Y151, which forms a hydrogen bond with the AA/ncAA backbone amino group (Kobayashi et al., 2003), may prevent AA binding. Finally, it may be that other AAs in the AA-binding pocket, such as H70, must be mutagenized to enable the efficient incorporation of aromatic ncAAs bearing meta or ortho substituents (Neumann et al., 2008; Sakamoto et al., 2009; Hauf et al., 2017).

Our results suggest that the aaRS variants analyzed in this study may also efficiently incorporate other phenylalanine derivatives that harbor 4' (para) substituents, which were not included in the current panel. It is also possible that some of these aaRS variants can efficiently incorporate one or a few instances of other aromatic ncAAs, including the remaining 23 ncAAs that we examined in this study and for which we did not identify an efficient aaRS for the incorporation of 10 or 30 instances per protein. However, the experimental conditions for the identification of such low-to-medium efficiency aaRS:ncAA pairs must be carefully selected, since the background expression of reporter protein in the absence of the ncAA can be high, and it increases with decreasing number of TAG codons per protein. Such analyses may be facilitated by decreasing aaRS production, for example by expression of a chromosomally integrated aaRS, or by reducing the copy number of the plasmid encoding the aaRS, or the amount of the respective inducer of aaRS expression (Amiram et al., 2015;Hadar et al., 2021). Finally, the high-performance aaRS:ncAA pairs identified in this study may also be utilized to improve the expression of ncAA-containing proteins and PBPs in other *E. coli* strains, such as derivatives of the commonly utilized BL21 strain, although competition with RF-1 is expected to reduce the yield of the full-length protein in these strains (Hadar et al., 2021).

### Multi-Site ncAA Incorporation in ELPs

The characterization of ncAA-containing ELPs requires a suitable ELP variant, such that ncAA incorporation will result in ELPs with measurable LCSTs (i.e., ∼10–90°C). However, the LCST of the above-mentioned ELP-GFP reporters is predicted to be either above (in GFP-fused proteins) or below (in unfused proteins) the measurable temperature range used for LCST analysis and characterization. Therefore, we selected a gene that encodes another ELP variant, termed ELP_60_(10TAG), which we previously utilized to determine the effect of the incorporation and isomerization of 5 on the ELP LCST (Israeli et al., 2021). The protein encoded by the ELP_60_(10TAG) gene is based on previously described hydrophilic ELPs, which have a high LCST (>90°C for a 25 μM solution (Meyer and Chilkoti, 2002)) and are composed of glycine and alanine, alternating in the X-guest residue position (Supplementary Table S3). We chose this variant since we hypothesized that the multi-site incorporation of aromatic ncAAs is expected to dramatically reduce the LCST of the ELP, based on the observation that the incorporation of aromatic residues, such as tyrosine, phenylalanine, tryptophan (Urry et al., 1992; Seifried et al., 2018), and 5 (Israeli et al., 2021), reduce the LCST, as compared with the incorporation of more hydrophilic AAs. Below, we name proteins according to the identity of the ncAA incorporated in the TAG codons. For example, ELP_60_(1 × 10) is the protein product of the ELP_60_(10TAG) gene, wherein 1 is incorporated in 10 encoded TAG codons.

**FIGURE 2:**
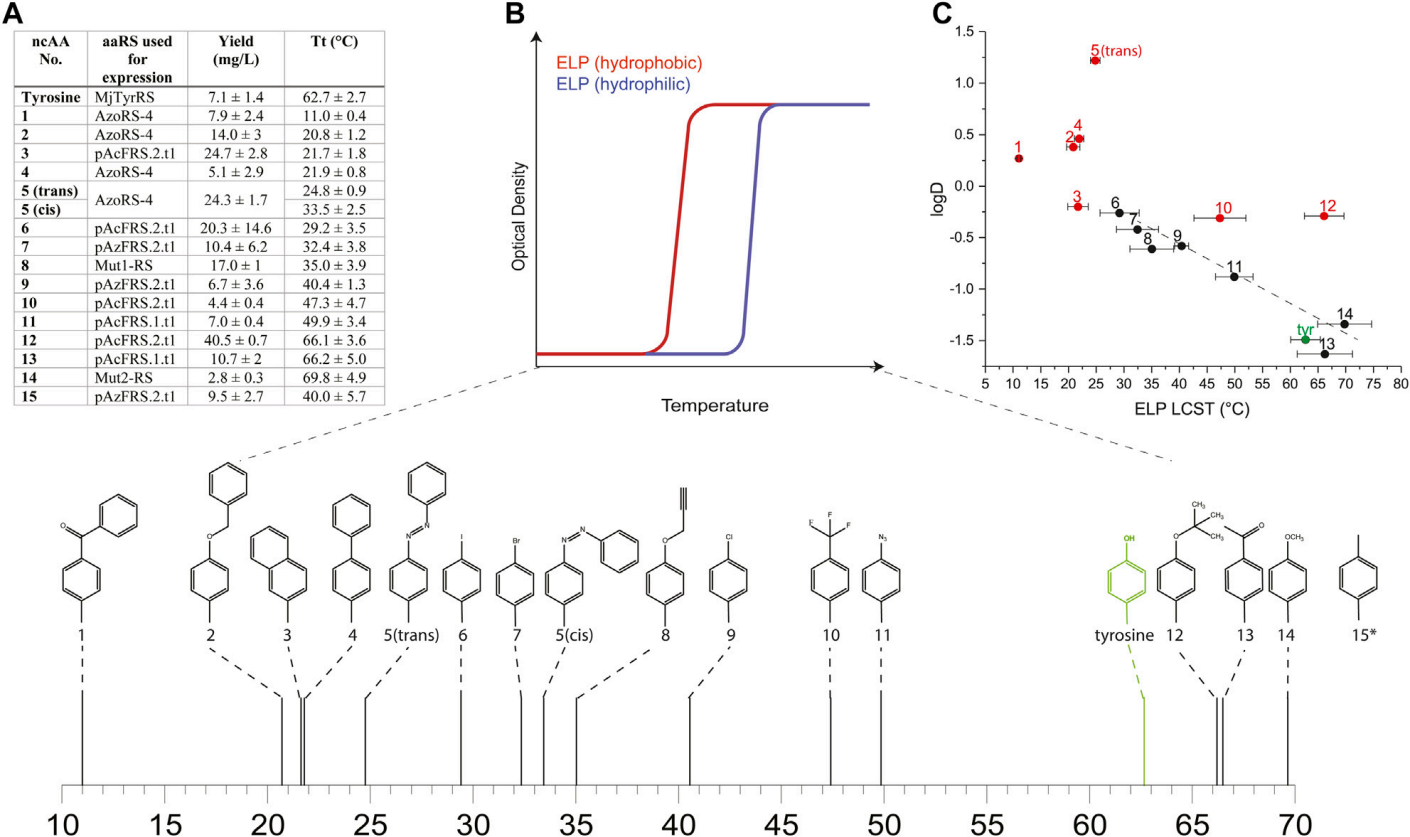
**(A)** Yields and LCST values calculated for ELPs containing each of the 15 ncAAs depicted in Supplementary Figure S1 and expressed using the indicated aaRS. **(B)** A schematic illustration of the effect of the relative hydrophobicity of the AA or ncAA incorporated into the ELP sequence on its LCST, and the measured LCST values for ELPs bearing ncAAs 1–14 (*the LCST of ELP_60_ (15 × 10) was too high to be measured in ddH_2_O, and was therefore determined in ddH_2_O supplemented with 1M NaCl). **(C)** The relationship between the LCST of ELPs bearing ncAAs 1–14 and the predicted logD values of the ncAAs. Red and black data points indicate ELPs that were found to self-assemble or that remained as monomers, respectively.

We expressed proteins from the ELP_60_(10TAG) gene in the C321. ΔRF1 *E. coli* strain by using either aaRSs suitable for each of the 15 ncAAs, or the native MjTyrRS (for the incorporation of tyrosine), as designated in Figure 2A. We quantified protein yields by using small-batch expression and found that the yields of ncAA-containing ELP_60_(10TAG) proteins ranged from ∼3 to 40 mg/L, as compared with the yield of tyrosine-containing ELP_60_(tyrosine×10), which was ∼7 mg/L (Figure 2A). The yield of purified proteins is likely affected both by the purification process, in which more hydrophobic ELPs generally tend to precipitate more easily, and by the efficiency of the aaRS. Protein purity and MW were determined by SDS-PAGE and intact mass-spectrometry (Supplementary Table S4 and Supplementary Figure S5).

To determine the effect of ncAA incorporation on ELP properties, we first examined the effect of ncAA identity on the LCST of the ELPs in ddH_2_O at a fixed concentration of 25 μM (Figures 2A,B, Supplementary Figure S6). The incorporation of 10 instances of each ncAA in the ELP_60_(10TAG) protein profoundly affected the LCST, which ranged from ∼11 to 70°C for ELP_60_(1 × 10)- ELP_60_(14 × 10), while the LCST of ELP_60_(15 × 10) was above 90°C in these conditions. We next examined the effect of ncAA incorporation on the self-assembly of this ELP family, motivated by an earlier finding that the incorporation of 5 engendered the self-assembly of ELP_60_(5 × 10) into thin 2-dimensional sheets. An assessment of self-assembly was conducted by a dynamic light scattering (DLS) analysis of a solution of each protein (25 μM in ddH_2_O) at a temperature below the LCST of all ELPs (5°C). The DLS analysis indicated that ncAAs 1–5, 10, 12, and 15 appeared to engender ELP self-assembly (Supplementary Figure S7). We note that previously reported ELP_60_(1 × 10) produced using a first-generation aaRS did not self-assemble, perhaps due to differences in mis-incorporation tendencies or in the purification protocol (Israeli et al., 2021). The effect of ncAA incorporation on the LCST of the ELPs can be viewed as an extension to Urry’s AA hydrophobicity scale, which is based on the effect of natural AAs on the ELP LCST (Urry et al., 1992). Therefore, we examined the relationship between the ELP LCST and predicted logD values of the ncAAs at pH 7 (computed using ChemAxon, which was previously shown to generate values that correlated well with the experimental logD measurements of AAs and similar ncAAs (Kubyshkin, 2021)). While a poor correlation was observed overall between the LCST of the ELPs and the computed logD values, a strong correlation (*R* ^2^ = 0.92) was observed for those ELPs that did not self-assemble in the solution (Figure 2C). This finding could be explained by the fact that self-assembly effectively alters the local concentration of the ELPs—and, therefore, its apparent LCST—as was suggested in other self-assembled ELP systems (Dreher et al., 2008; Prhashanna et al., 2019).

Next, we examined the secondary structure of ncAA-containing ELPs by using circular dichroism (CD) spectroscopy at a temperature below the LCST of all ELPs (5°C). All ELPs, including the control ELP_60_(Tyrosine×10), showed negative peaks at around ∼190 nm, and most ELPs exhibited an additional negative peak at ∼220 nm; these peaks are characteristic of random-coil and β-turn structures, respectively (Supplementary Figure S8) (Janib et al., 2014). The magnitude and ratio of these peaks varied for each ELP, indicating that ncAA incorporation can also affect the secondary structure of these proteins. However, no correlation was found between these structural properties, the LCST, or the self-assembly propensity of the ELPs, as determined in these conditions.

Precise tuning of the properties, such as the LCST, of ELPs (and other PBPs), either as single- or multi-block polymers or as fusions with other proteins and PBPs (e.g., ELP–RLP fusions), is required in many applications (McDaniel et al., 2013), such as for drug delivery (Bhattacharyya et al., 2016; Varanko et al., 2020; Balu et al., 2021), tissue engineering (Bhattacharyya et al., 2016; Balu et al., 2021), sensing (Dhandhukia et al., 2013; Dhandhukia et al., 2017), metabolic engineering (Dzuricky et al., 2020), and protein purification (Hassouneh et al., 2010; Bhattacharyya et al., 2016). Although factors such as salinity and MW can also be used to tune the LCST or UCST of PBPs, such adjustments are often limited by the specific application (McDaniel et al., 2013). Previous studies have demonstrated that either installing or incorporating unnatural chemical groups can modulate ELP properties, such as LCST. For example, a global replacement of proline residues with various proline analogs in ELPs resulted in changes to the secondary structure of the ELPs and altered their LCST (Kim et al., 2005; Kim et al., 2006; Catherine et al., 2015). Similarly, the chemical modification of methionine residues encoded in the guest-residue position in ELPs also enabled the tuning of the LCST (Kramer et al., 2015; Petitdemange et al., 2017; Rosselin et al., 2019) and triggered the self-assembly of di-block ELPs (Dai et al., 2021). However, until recently, the moderate efficiency of orthogonal translation systems has limited the production of PBPs that contain multiple instances of ncAAs by genetic code expansion, and thereby hindered the analysis of the resulting properties (Wang et al., 2021).

Here, we demonstrate that high-performance aaRSs enable the incorporation of 15 different aromatic ncAAs in ELPs—and, in turn, the production of a family of ELPs with LCSTs that vary within a wide temperature range—from a single DNA template. In addition, the analysis of these ELPs revealed that ncAA incorporation affects the secondary structure and self-assembly of the ELPs. Importantly, as compared with global replacement or chemical modification, which modify every instance of a particular AA, incorporating ncAAs using high-performance orthogonal translation components enables the multi-site incorporation of the selected unnatural side-chain alongside the entire set of the 20 natural AAs. The distinct advantage of this approach is particularly valuable in the design of ELP-fusion proteins wherein unintended AA modifications can result in suboptimal PBP properties or reduce or abolish the activity of the fused protein (Costa et al., 2019; Vanderschuren et al., 2022).

### Multi-Site ncAA Incorporation in RLPs

While a few previous studies have reported the effects of ncAA incorporation or side-chain modifications on the properties and behaviors of ELPs (Kim et al., 2005; Kim et al., 2006; Catherine et al., 2015; Kramer et al., 2015; Petitdemange et al., 2017; Rosselin et al., 2019), we are not aware of any similar analyses of such alterations in other PBPs. To examine the effect of ncAA incorporation on the UCST-type phase transition behavior, we turned our attention to a recently described family of RLPs constructed from tandem repeats of the GRGDSPYS peptide (Dzuricky et al., 2020). We produced a series of proteins from the GRGDSPYS_40_(6TAG) gene (Supplementary Table S3), with either tyrosine (which results in the original “wild type” sequence) or ncAAs 1–15 encoded in the TAG codons. Protein purity and MW were determined by SDS-PAGE and intact mass-spectrometry (Supplementary Table S5 and Supplementary Figure S9).

**FIGURE 3:**
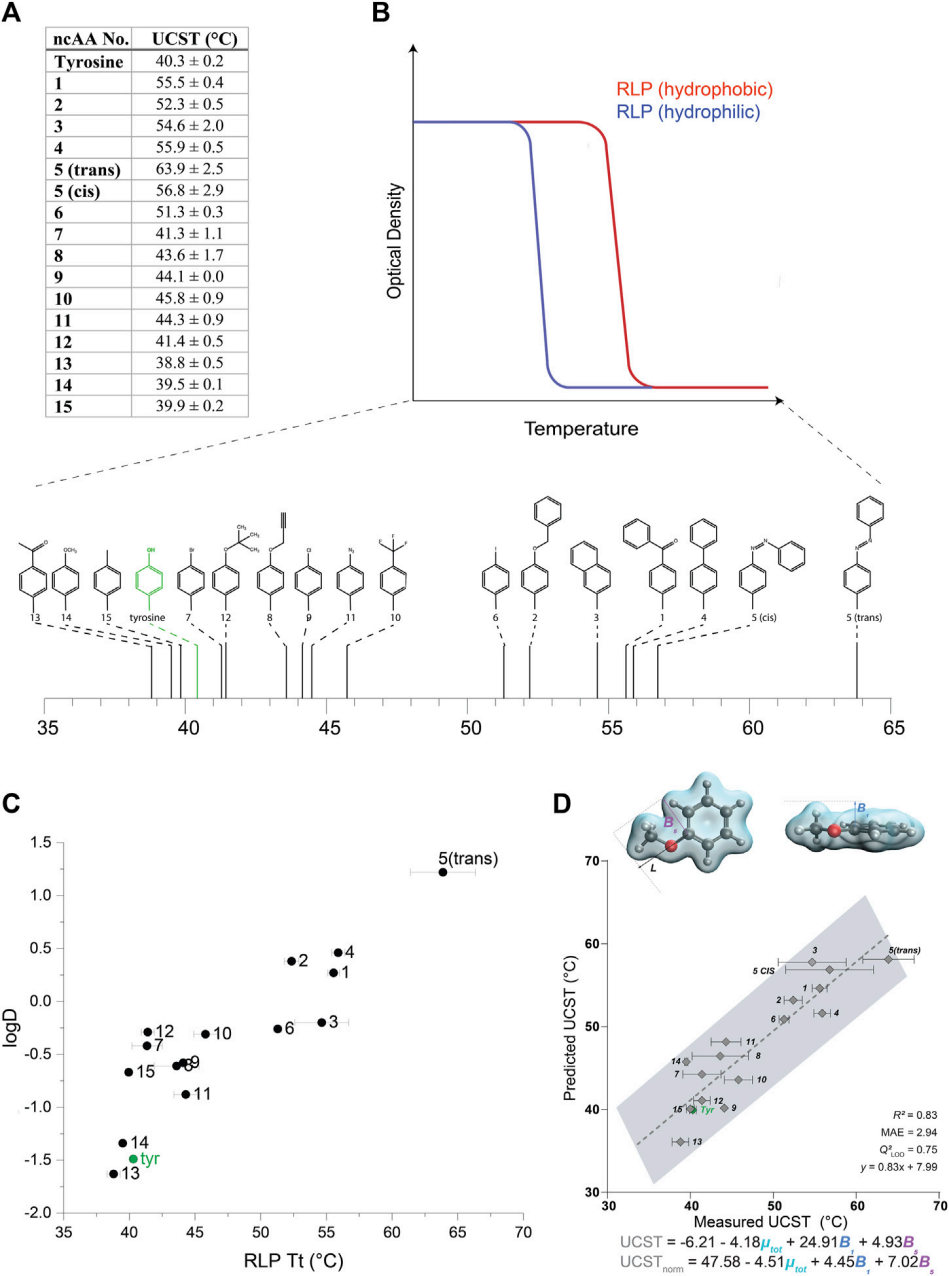
**(A)** UCST values calculated for RLPs containing each of the 15 ncAAs depicted in Supplementary Figure S1. **(B)** A schematic illustration of the effect of the relative hydrophobicity of the AA or ncAA incorporated in the RLP sequence on its UCST, and a schematic indicating the measured UCST values for the RLPs bearing ncAAs 1–15. **(C)** The relationship between the UCST of RLPs bearing ncAAs 1–15, and predicted logD values of the ncAAs. **(D)** Multivariate linear regression model that includes *B*_1_, *B*_5_, and *µ*-total as predictive molecular descriptors for the aggregation temperature of different RLP analogs. The goodness-of-fit is indicated by *R* ^2^ and Q^2^ for a leave-one-one (LOO) cross validation. The equations predicting the transition temperature, UCST and USCT_norm_, are indicated for raw and normalized parameters (by subtracting the respective mean and dividing by the standard deviation), respectively.

We then examined the effect of the different ncAAs on the UCST of this set of RLPs (Figures 3A,B, Supplementary Figure S10). The incorporation of more hydrophilic ncAAs, as estimated by predicted logD values, typically resulted in a reduction of the UCST, such that the solubilization of the protein occurred at lower temperatures (Figures 3A,B) (we note that all the RLP variants appeared to self-assemble above their UCST, Supplementary Figure S11). As expected, this effect was generally opposite to that displayed by the ELPs, wherein the more hydrophilic ncAAs increased the LCST, such that the aggregation of the protein occurred at higher temperatures. Indeed, the correlation (*R* ^2^ = 0.77) between the predicted logD values and the UCST of the RLPs indicated that increasing ncAA hydrophobicity generally increases the UCST of the RLP (Figure 3C).

Although hydrophobic interactions broadly explain the aggregation of different RLP analogs at various temperatures, it is well established that other interactions (e.g., π-π, cation-π, hydrogen bonds) also strongly affect the UCST. Therefore, we were interested in utilizing the data collected on this large set of aromatic substitutions to gain a more in-depth understanding of their effect on the phase-transition behavior at the molecular level. To this end, a multivariate model was identified to elucidate the specific molecular features of the RLP derivatives that may influence their UCST. Based on the versatile modifications on the aromatic ring of the tyrosine side chain, we evaluated the steric, electronic, and stereo-electronic molecular descriptors, such as Sterimol parameters (Verloop et al., 1976), vibration frequencies and intensities (Milo et al., 2014), dipole moment and NBO charges (Glendening et al., 2012) (Supplementary Appendix 1). The best-fitted model included the total dipole moment of the side-chain and two steric Sterimol parameters, B1 and B5, representing the minimal and maximal width of the substituent, respectively (Figure 3D). Overall, wider analogs with decreased polarity, reflected as larger B1 and B5 values and a smaller total dipole variable, led to an increased transition temperature. We speculate that the decreased polarity could reflect hydrophobicity, whereas the width parameters could represent steric hindrance. These results suggest a decreased transition temperature for both more hydrophilic residues and for less sterically hindered substituents that do not impede hydrogen bonding with the aqueous solution.

Several studies demonstrated that the number, position, and identity of aromatic AAs have a significant effect on the UCST of RLPs and other natural and artificial intrinsically disordered proteins\regions (IDP\Rs) known to exhibit UCST-type behavior. Specifically, an extensive characterization of RLPs composed of the “GRGDSPYS” motif, and the effect of substitutions of tyrosine residues to other aromatic or non-aromatic AAs, revealed that the UCST was substantially effected by the identity of the aromatic AA in the repeat sequence, and by an arginine-to-lysine substitution, which affects cation-π interactions with the aromatic AAs (Dzuricky et al., 2020). Here, we show that substituting only six of the 40 tyrosine residues in the repeating GRGDSPYS_40_ protein sequence can generate a difference of ∼25°C in the UCST of these PBPs. Likewise, it is expected that ncAA incorporation will enable the tuning of the UCST of other PBPs and IDP/Rs whose properties can be altered by aromatic AAs. For example, mutations of aromatic and positively charged AAs have been shown to modulate the UCST of several natural IDP/Rs, such as proteins derived from the Fused in Sarcoma (FUS) family (Lin et al., 2017; Wang et al., 2018), IDR regions of Ddx4 (Nott et al., 2015; Brady et al., 2017), and the intrinsically disordered RGG domain of the LAF-1 protein (a component of P granules) (Schuster et al., 2020). We expect that the ability to incorporate multiple instances of various aromatic ncAAs in these and many other PBPs and IDPs will continue to inform the molecular-level behavior of their phase transition and enable the rational tuning of their UCSTs and, thereby, also of their self-assembly propensities.

## Conclusion

We report a suit of high-performance aaRSs for the multi-site incorporation of 15 different aromatic ncAAs. Furthermore, our comparative analysis of 11 aaRS variants that evolved from a single ancestor (MjTyrRS), using an identical mutagenesis strategy, enabled the analysis of the mutational landscape at the ncAA-binding site which can inform future aaRS evolution efforts. This analysis indicates a distinct pattern of mutations found in the ncAA-binding site and shows that the mutagenesis of only nine of the 12 residues targeted in our library designs may suffice for the selection of efficient aaRS variants for the incorporation of para-substituted aromatic ncAAs. The application of this knowledge toward the creation of more focused libraries will enable the exploration of a larger subset of the relevant theoretical library space, and thus the selection of more efficient variants (Hauf et al., 2017; Baumann et al., 2019). Employing these capabilities, we were able to provide the first investigation of the effect of various unnatural aromatic groups on the ELP LCST- and RLP UCST-type phase-transition behaviors. We show that the incorporation of aromatic ncAAs can generate, from a single DNA template, a family of ELP proteins that have a wide range (spanning >60°C) of LCSTs. Further analysis of the ELP properties reveals that ncAA incorporation affects both the secondary structure of the ELPs and, for some ncAAs, the propensity to self-assemble. Moreover, we show that aromatic ncAA incorporation can also be utilized to tune the phase-transition behavior of RLPs, and that this behavior is strongly affected by both the hydrophobicity and the size of the ncAA side-chain. Given the recognized importance of aromatic residues for the phase transition behavior—primarily of the UCST-type, but also of the LCST-type—the ability to efficiently encode aromatic ncAAs alongside the entire set of 20 natural AAs will allow access to new families of precise and chemically diverse PBPs and PBP-protein fusions. Expanding the chemical diversity of aromatic side chains incorporated in ELP- and RLP-based PBPs will permit further investigation of the effect of aromatic character on the different phase-transition behaviors and advance our understanding of the functions and sequence determinants of LCST- and UCST-type phase transitions in natural and artificial IDPs and PBPs (Ruff et al., 2018). Beyond the specific families explored in this work, these capabilities will empower future studies of the effect of ncAA incorporation on the self-assembly propensities and morphologies of multi-block polymers composed of domains derived from ELPs, RLPs, or other PBPs. We expect that further elucidation of the sequence and molecular determinants of these and many other PBPs, IDP/Rs, and ncAAs will enable the rational design of semi-synthetic polymers with bespoke new or improved properties.

## Data Availability

The original contributions presented in the study are included in the article/Supplementary Material, further inquiries can be directed to the corresponding author.
