# Supplementary material for: Tuning the Properties of Protein-Based Polymers Using High-Performance Orthogonal Translation Systems for the Incorporation of Aromatic Non-Canonical Amino Acids
Source: Front Bioeng Biotechnol. 2022 May 30;10:913057. doi: 10.3389/fbioe.2022.913057 (PMC9195583; doi:10.3389/fbioe.2022.913057)
Supplement: Supplementary file 1 [file DataSheet1.pdf]

Table S1. ncAAs used in this study

name	Identifier	CAS	Vendor
4-Benzoyl-L-phenylalanine	1	104504-45-2	Chemimpex
O-Benzyl-L-tyrosine	2	16652-64-5	Chemimpex
3-(2-Naphthyl)-L-alanine	3	58438-03-2	Chemimpex
H-p-Phenyl-L-phenylalanine	4	155760-02-4	Chemimpex
phenylalanine-4‘-azobenzene	5	2137036-84-9	Giotto Biotech
4-Iodo-L-phenylalanine	6	24250-85-9	Chemimpex
4-Bromo-L-phenylalanine	7	24250-84-8	Chemimpex
4-propargyloxy-L-phenylalanine	8	1080496-42-9	Chemimpex
4-Chloro-L-phenylalanine	9	14173-39-8	Chemimpex
p-Trifluoromethyl-L-phenylalanine	10	114926-38-4	Chemimpex
P-azido-L-phenylalanine	11	33173-53-4	Iris Biotech
O-tert-Butyl-L-tyrosine	12	18822-59-8	Chemimpex
P-acetyl-L-phenylalanine	13	122555-04-8	Santa Cruz Biotechnology
O-Methyl-L-tyrosine	14	6230-11-1	Chemimpex
4-Methyl-L-phenylalanine	15	1991-87-3	Chemimpex
3-Nitro-L-tyrosine	16	621-44-3	Chemimpex
4-Amino-L-phenylalanine	17	943-80-6	Chemimpex
3-Amino-L-tyrosine	18	23279-22-3	Chemimpex
2-Nitro-L-phenylalanine	19	19883-75-1	Chemimpex
3-Cyano-L-phenylalanine	20	144799-02-0	Chemimpex
O-Acetyl-L-tyrosine	21	6636-22-2	Chemimpex
4-Cyano-L-phenylalanine	22	104531-20-6	Chemimpex
2-Cyano-L-phenylalanine	23	263396-42-5	Chemimpex
2-Methyl-L-phenylalanine	24	80126-53-0	Chemimpex
3-Chloro-L-phenylalanine	25	80126-51-8	Chemimpex
3-Chloro-L-tyrosine	26	7423-93-0	Chemimpex
2-Chloro-L-phenylalanine	27	103616-89-3	Chemimpex
3-Fluoro-L-phenylalanine	28	19883-77-3	Chemimpex
4-Fluoro-L-phenylalanine	29	1132-68-9	Chemimpex
2,6-Difluoro-L-phenylalanine	30	33787-05-2	Chemimpex
2,4-Difluoro-L-phenylalanine	31	31105-93-8	Chemimpex
3,4,5-Trifluoro-L-phenylalanine	32	646066-73-1	Chemimpex
2-Fluoro-L-phenylalanine	33	19883-78-4	Chemimpex
3-Trifluoromethyl-L-phenylalanine	34	14464-68-7	Chemimpex
3-Iodo-L-phenylalanine	35	20846-39-3	Chemimpex
2-Bromo-L-phenylalanine	36	42538-40-9	Chemimpex
2-Iodo-L-phenylalanine	37	167817-55-2	Chemimpex
3-Fluoro-L-tyrosine	38	7423-96-3	Chemimpex

Table S2. Extinction coefficients (measured at 280 nm) of ncAAs and proteins used in this study

ncAA identifier	ncAA extinction [$M^{-1} \cdot cm^{-1}$]	ELP_{60}(10TAG) extinction [$M^{-1} \cdot cm^{-1}$]	$GRGDSPYS_{40}$(6TAG) extinction [$M^{-1} \cdot cm^{-1}$]
**1**	11087	112360	118672
**2**	1131	12802	58937
**3**	3638	37873	73980
**4**	2232	23810	65542
**5**	2541	26900	67396
**6**	321	4698	54075
**7**	75	2241	52600
**8**	1200	13490	59350
**9**	47	1957	52430
**10**	4	1532	52175
**11**	2728	28770	68518
**12**	120	2687	52868
**13**	3379	35280	72424
**14**	1044	11933	58416
**15**	32	1806	52340
**tyrosine**	1490	16390	61090

Table S3. PBP sequences used in this study

Protein	Amino acid sequence (* denotes the TAG codon)
ELP(10TAG)-GFP	SKGPG(VPGGGVPGAGVPG*G)$_{10}$PGGGG-(GFP-WT)
ELP(10TAG)-GFP	SKGPG(VPGGGVPGAGVPG*G)$_{30}$PGGGG-(GFP-WT)
ELP_{60}(10TAG)	G[(VPGGGVPGAG)$_2$(VPGGG VPG*G)]$_{10}$GY
$GRGDSPYS_{40}$(6TAG)	MGHHHHHHGHHHHHH [(GRGDSPYSGRG DSPYSGRGDS PYSGRGDSPY SGRGDSPYSG RGDSP *S)$_3$(GRGDSPYSG RGDSPYS)]$_2$GGY

Table S4. Intact mass measurements of ELPs produced in this study

ELP variant	Expected (g/mol)	Measured (g/mol)	Error (%)
ELP_{60}(**tyrosine**×10)	23681.37	23664.686	0.07%
ELP_{60}(**1**×10)	24564.37	24538.359	0.11%
ELP_{60}(**2**×10)	24584.37	24564.966	0.08%
ELP_{60}(**3**×10)	24024.37	24003.028	0.09%
ELP_{60}(**4**×10)	24284.27	24284.615	0.00%
ELP_{60}(**5**×10)	24,562.47	24519.49	0.17%
ELP_{60}(**6**×10)	24782.37	24753.166	0.12%
ELP_{60}(**7**×10)	24312.37	24211.414	0.42%
ELP_{60}(**8**×10)	24063.77	24046.719	0.07%
ELP_{60}(**9**×10)	23867.37	23836.603	0.13%
ELP_{60}(**10**×10)	24203.27	24179.846	0.10%
ELP_{60}(**11**×10)	23932.37	23854.206	0.33%
ELP_{60}(**12**×10)	24244.37	24213.531	0.13%
ELP_{60}(**13**×10)	23943.67	23935.026	0.04%
ELP_{60}(**14**×10)	23823.57	23807.107	0.07%
ELP_{60}(**15**×10)	23663.67	23651.468	0.05%

Table S5. Intact mass measurements of RLPs produced in this study

ELP variant	Expected (g/mol)	Measured (g/mol)	Error (%)
$GRGDSPYS_{40}$-(**tyrosine**×6)	34848.25	34774.83	0.21%
$GRGDSPYS_{40}$-(**1**×6)	35378.05	35429.42	-0.15%
$GRGDSPYS_{40}$-(**2**×6)	35390.05	35350.24	0.11%
$GRGDSPYS_{40}$-(**3**×6)	35054.05	35032.58	0.06%
$GRGDSPYS_{40}$-(**4**×6)	35209.99	35207.93	0.01%
$GRGDSPYS_{40}$-(**5**×6)	35,376.91	35,402.83	-0.07%
$GRGDSPYS_{40}$-(**6**×6)	35508.85	35496.37	0.04%
$GRGDSPYS_{40}$-(**7**×6)	35226.85	35072.32	0.44%
$GRGDSPYS_{40}$-(**8**×6)	35077.69	35047.06	0.09%
$GRGDSPYS_{40}$-(**9**×6)	34959.85	34900.86	0.17%
$GRGDSPYS_{40}$-(**10**×6)	35161.39	35123.59	0.11%
$GRGDSPYS_{40}$-(**11**×6)	34998.85	34964.47	0.10%
$GRGDSPYS_{40}$-(**12**×6)	35186.05	35212.91	-0.08%
$GRGDSPYS_{40}$-(**13**×6)	35005.63	34965.77	0.11%
$GRGDSPYS_{40}$-(**14**×6)	34933.57	34943.56	-0.03%
$GRGDSPYS_{40}$-(**15**×6)	34837.63	34865.95	-0.08%

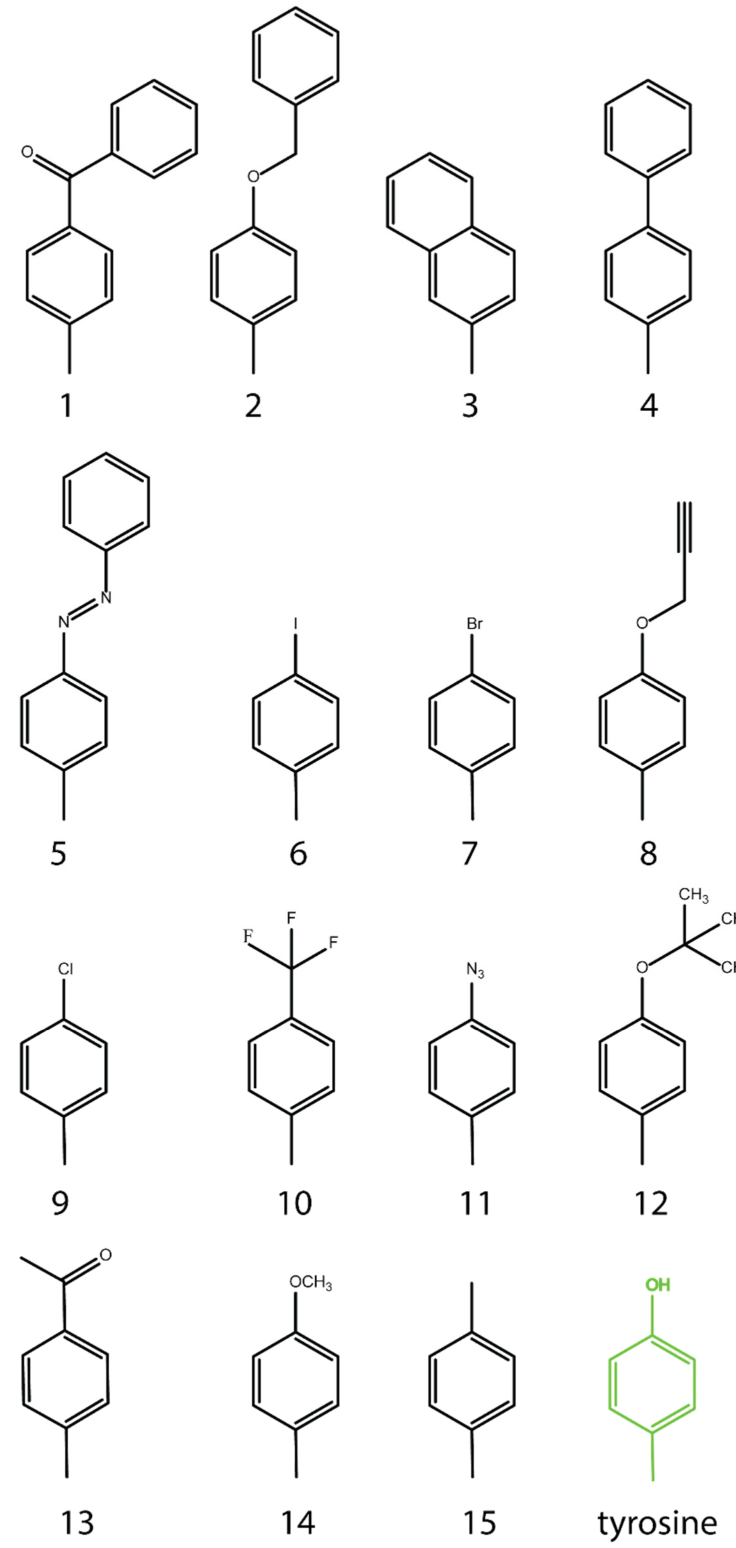

Figure S1. Chemical structures of the aromatic side chains of ncAAs that were successfully incorporated using the aaRS variants tested in this study and tyrosine (control).

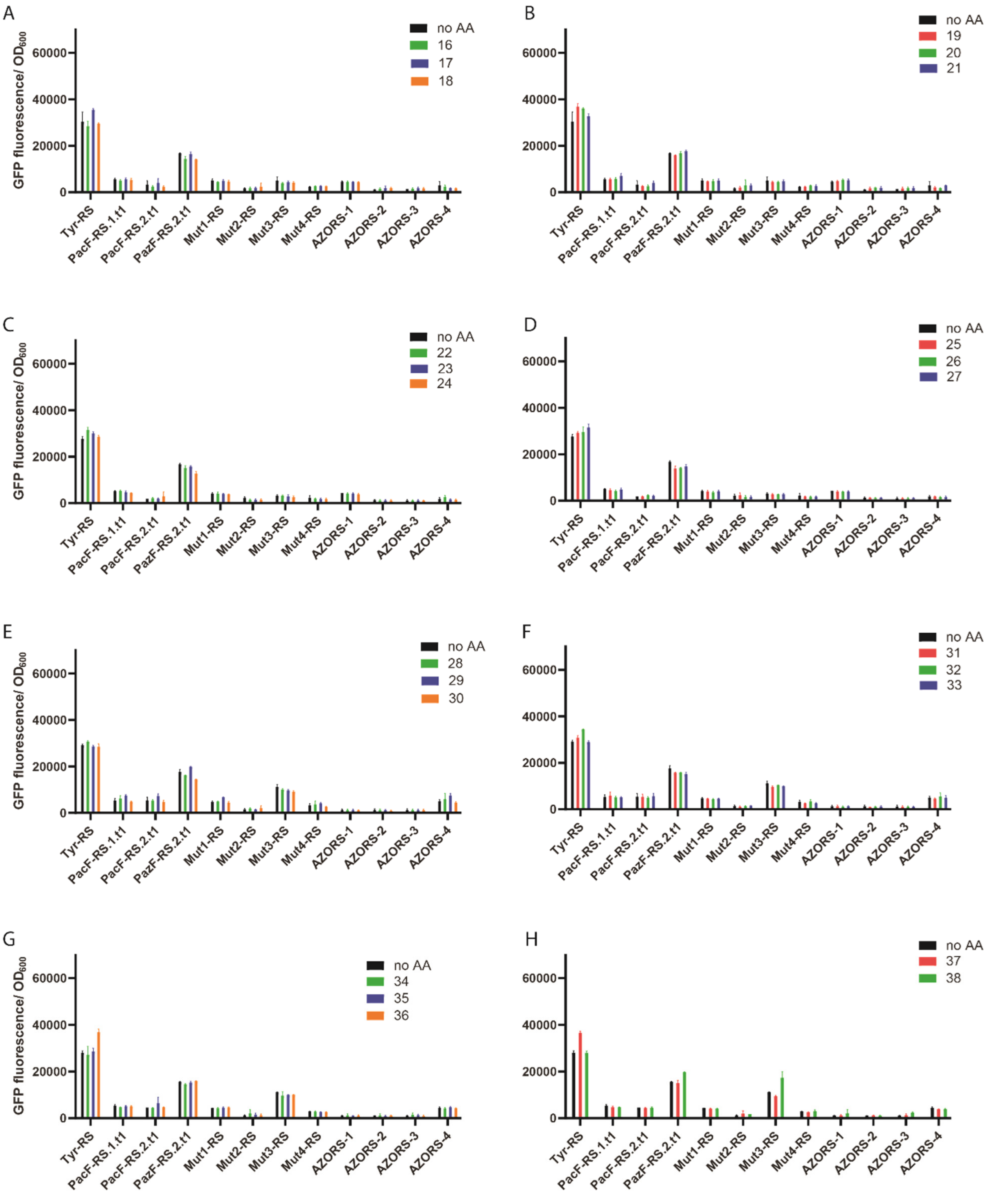

Figure S2. Activity analysis, using the reporter protein ELP(30TAG)-GFP, of high-performance aaRS variants for ncAAs **16-38** which are not efficiently incorporated by any of the tested aaRS variants.

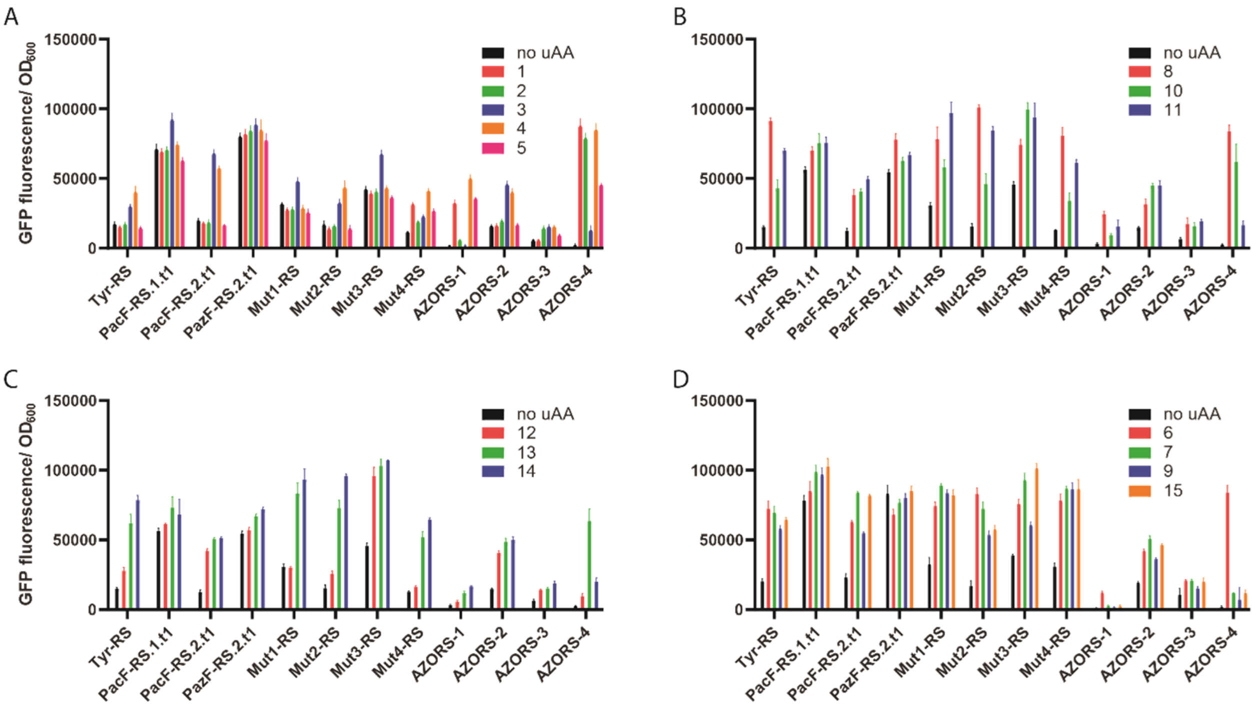

Figure S3. Specificities of high-performance aaRS variants for 15 ncAAs which are efficiently incorporated in the expression of the reporter protein ELP(10TAG)-GFP.

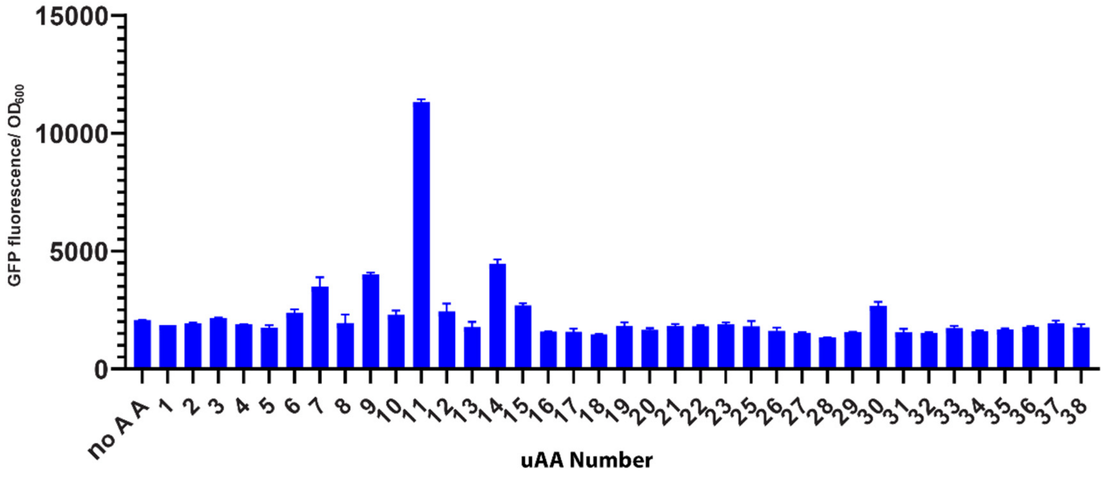

Figure S4. Specificity of the high-performance aaRS variant pAzFRS.1.t1 for the incorporation of pAzF (ncAA **11**) in the expression of the reporter protein ELP(30TAG)-GFP.

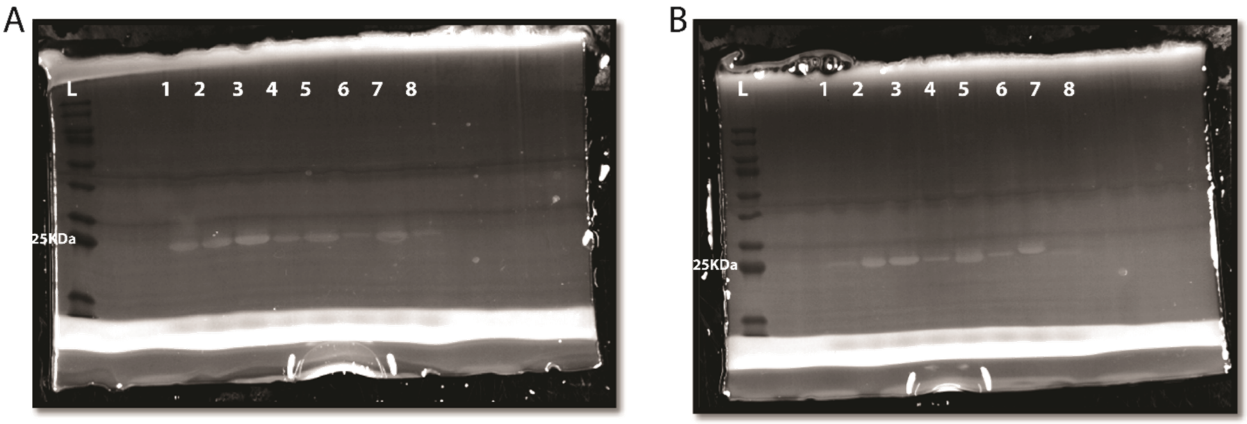

Figure S5. SDS-PAGE of ELP proteins described in this study stained with zinc stain to better visualize some of the ELP proteins which are poorly stained by Coomassie. (A) L: protein ladder, 1: ELP_{60}(**7**×10), 2: ELP_{60}(**9**×10), 3: ELP_{60}(**8**×10), 4: ELP_{60}(**6**×10), 5: ELP_{60}(**3**×10), 6: ELP_{60}(**2**×10), 7: ELP_{60}(**1**×10), 8: ELP_{60}(**5**×10). (B) L: protein ladder, 1: ELP_{60}(**10**×10), 2: ELP_{60}(**14**×10), 3: ELP_{60}(**13**×10), 4: ELP_{60}(**15**×10), 5: ELP_{60}(**11**×10), 6: ELP_{60}(**12**×10), 7: ELP_{60}(**tyrosine**×10), 8: ELP_{60}(**4**×10).

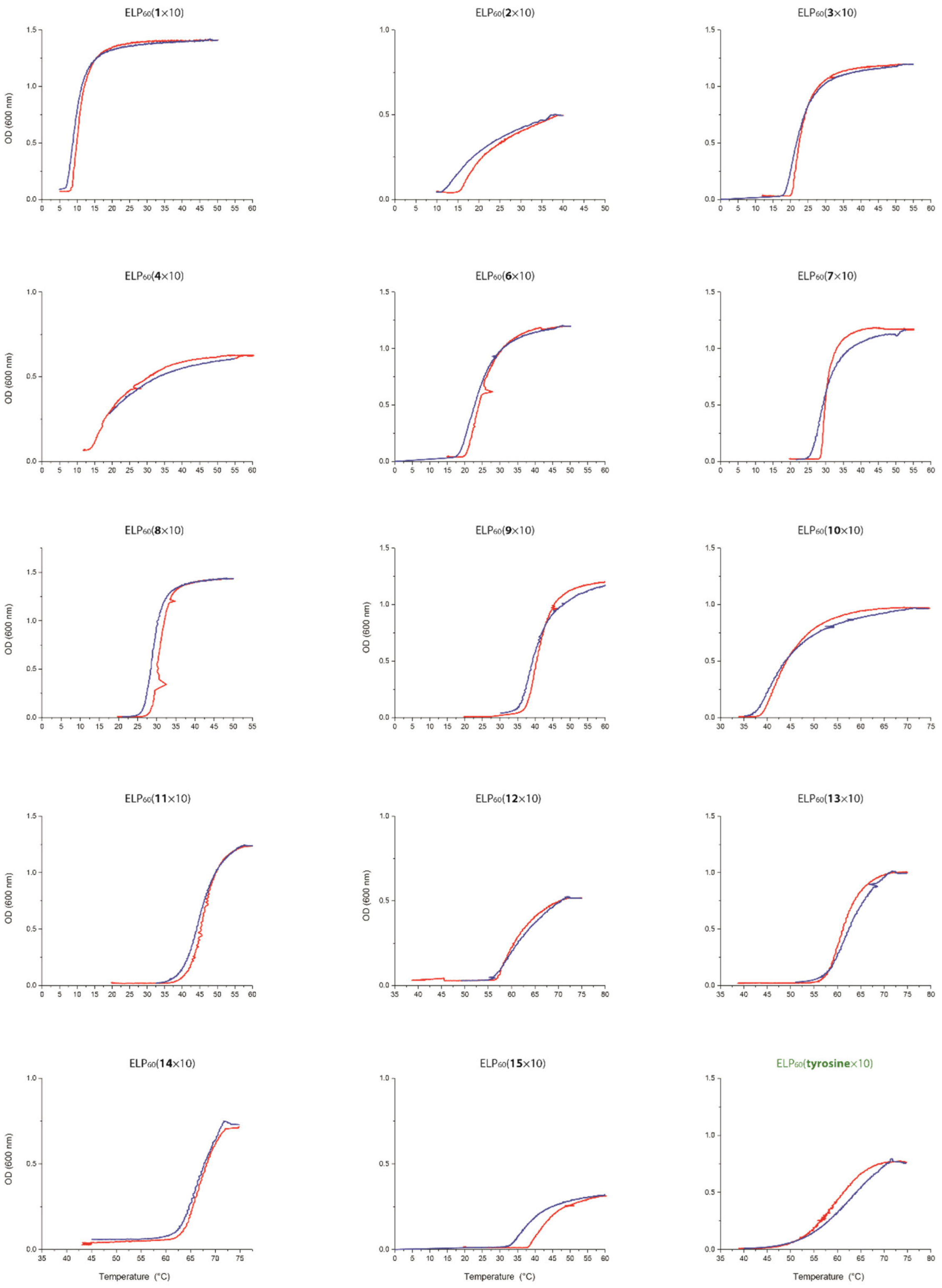

Figure S6: Representative turbidity profiles as a function of temperature for ELP proteins used in this study (25μM in ddH_2O for all proteins except for ELP_{60}(**15**×10) which was supplemented with 1 M NaCl). Red curves – heating, blue curves – cooling.

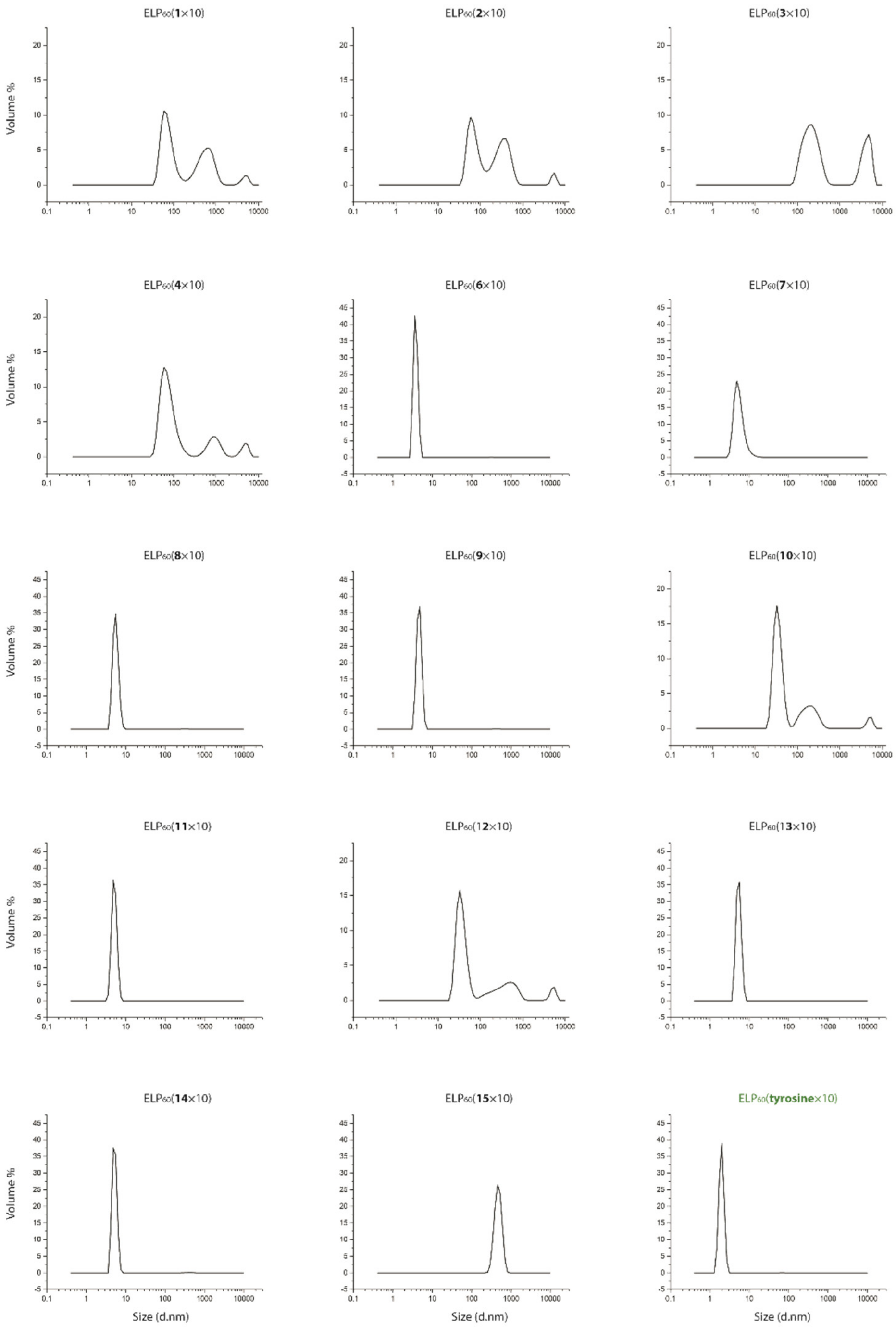

Figure S7: Dynamic light scattering analysis of ELP proteins introduced in this study (25μM in ddH_2O at 5 °C)

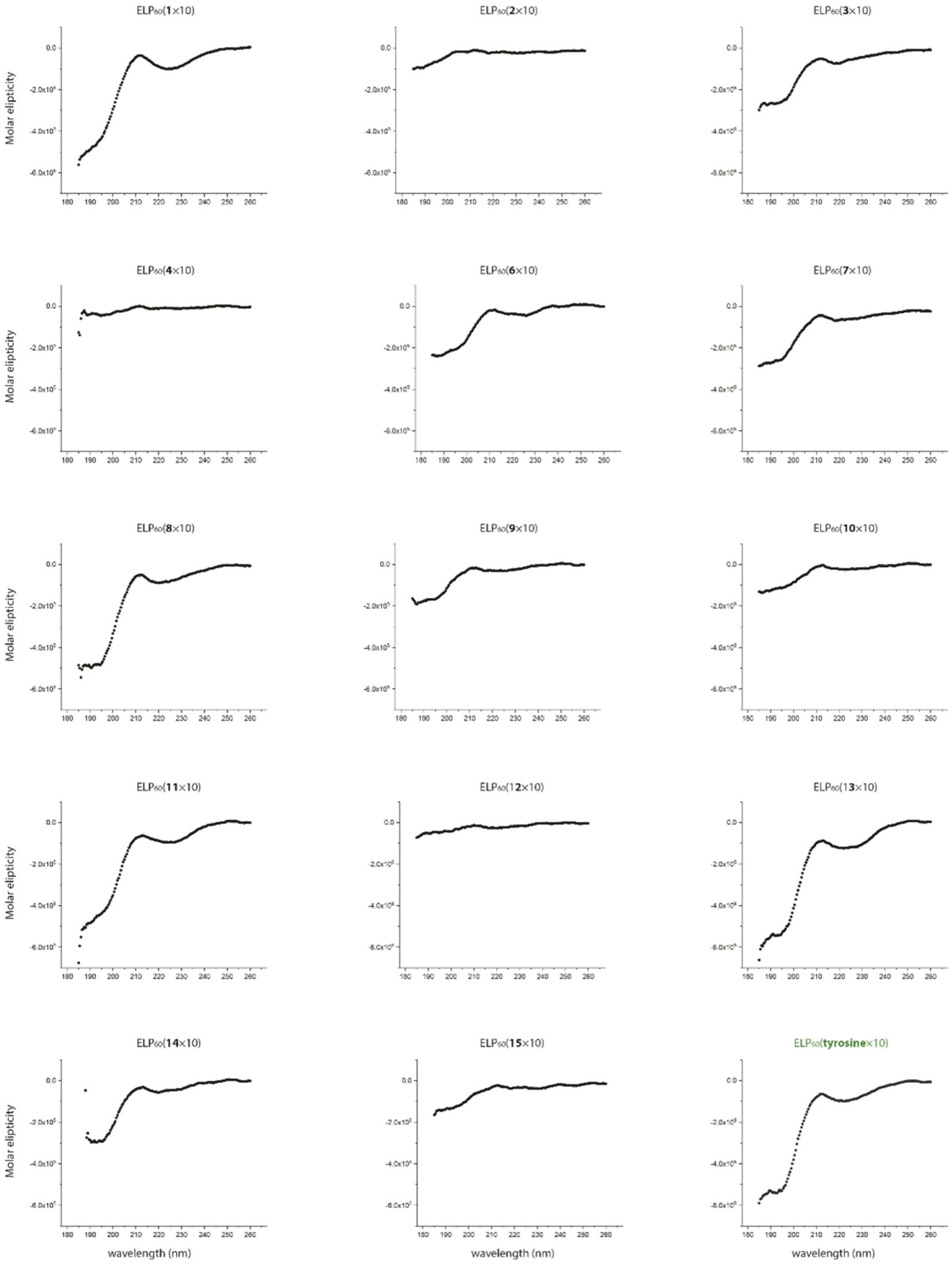

Figure S8: Comparison of the CD spectra of ELP proteins introduced in this study (25μM in ddH_2O at 5 °C)

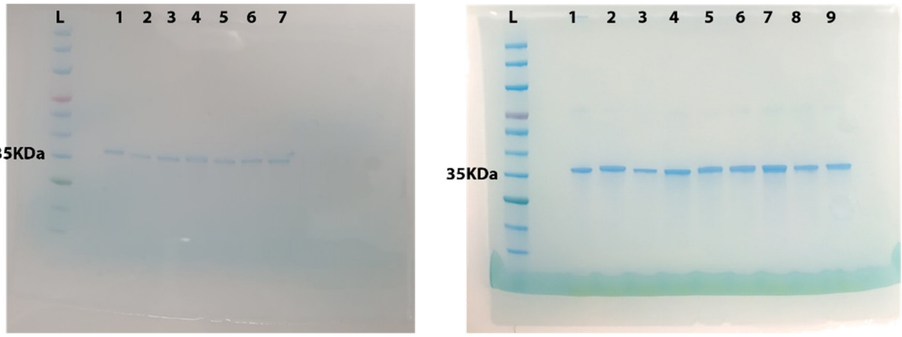

Figure S9: SDS-PAGE of resilin-inspired proteins described in this study stained with InstantBlue™ Coomassie stain. (A) L: protein ladder, 1: GRGDSPYS$_{40}$(**tyrosine**×6), 2: GRGDSPYS$_{40}$(**2**×6), 3: GRGDSPYS$_{40}$(**8**×6), 4: GRGDSPYS$_{40}$(**11**×6), 5: GRGDSPYS$_{40}$(**5**×6), 6: GRGDSPYS$_{40}$(**1**×6), 7: GRGDSPYS$_{40}$(**10**×6). (B) L: protein ladder, 1: GRGDSPYS$_{40}$(**6**×6), 2: GRGDSPYS$_{40}$(**9**×6), 3: GRGDSPYS$_{40}$(**4**×6), 4: GRGDSPYS$_{40}$(**2**×6), 5: GRGDSPYS$_{40}$(**7**×6), 6: GRGDSPYS$_{40}$(**15**×6), 7: GRGDSPYS$_{40}$(**14**×6), 8: GRGDSPYS$_{40}$(**12**×6), 9: GRGDSPYS$_{40}$(**13**×6).

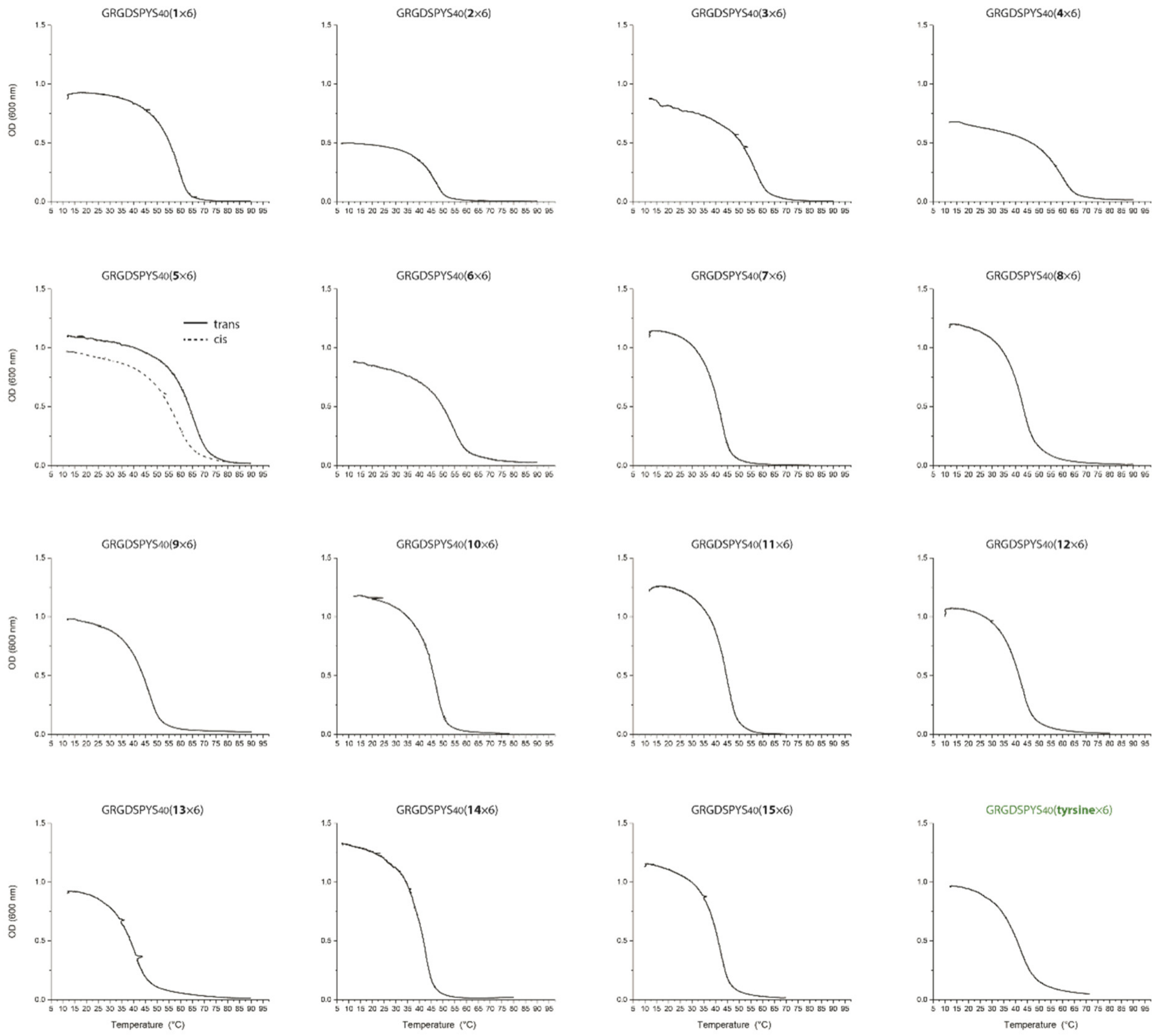

Figure S10: Representative turbidity profiles as a function of temperature for RLP proteins used in this study (25μM in PBS supplemented with 0.5 M Urea).

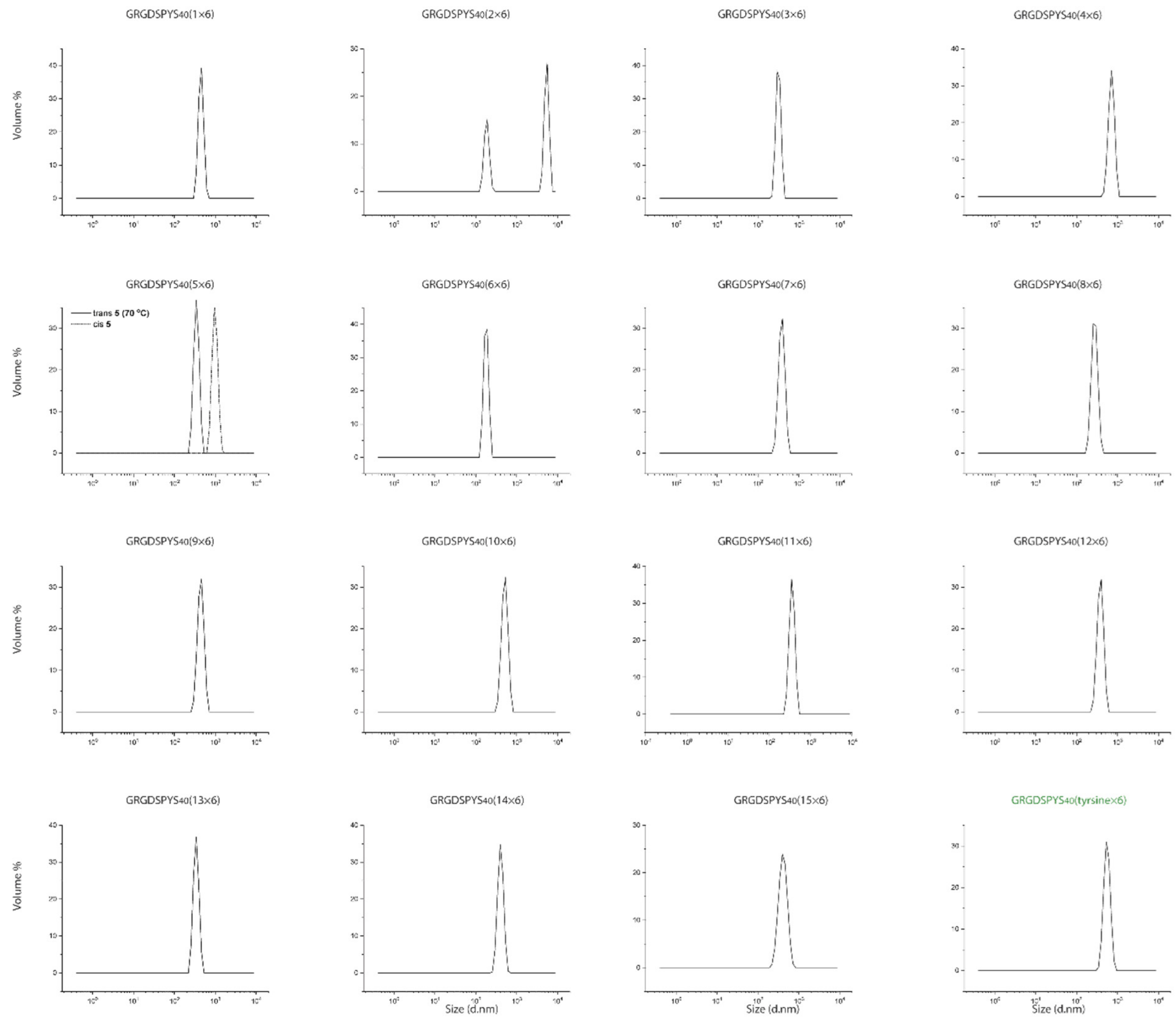

Figure S11: Dynamic light scattering analysis of RLP proteins used in this study (25μM in PBS supplemented with 0.5 M Urea at 60 °C unless otherwise indicated).

<u>**Appendix 1. Model development**</u>

1. DFT computations

All DFT calculations were carried out using Gaussian 16 software.[1] The functional used for Geometry optimizations and frequency calculations is M06-2X, which was previously benchmarked for thermodynamic and kinetic accuracy of main group elements, and for non-covalent interactions.[2,3] Based on Zhao and Truhlar's evaluation of the M06-2X functional for organic molecules, indicating that a triple zeta quality is generally more quantitative,[5] a triple zeta potential basis-set (def2-TZVP) was chosen. [4,5] Since this study seeks correlations, we opted not to incorporate scaling factors for vibrational terms.[6] This simplification is justified by the assumption that a constant scaling factor would neither change the descriptive parameters identified, nor the relationship between them.

2. Parameterization

Sterimol values were calculated for all geometry optimized structures with the use of our own in-house developed Sterimol program (available at https://github.com/Milo-group/SteRimol), based on Verloop's original definitions.[7] The values were calculated along the substituent axis L, such that the principal axis begins on the substituent connected to the aromatic moiety. Thus, L also represents the length of the substituents on the aromatic side-chain of the tyrosine. B_5 and B_1, representing the maximal width and the minimal width perpendicular to L, respectively. The origin of the dipole moment μ parameter was set to the bond of the substituent to the aromatic ring. The dipole moment μ parameter was calculated for the geometry optimized structures based on Gaussian's default dipole computation with respect to a consistent Cartesian origin located on the bond of the substituent on the aromatic ring. Bond and ring vibrational frequencies, bond lengths, angles and distances between atoms were extracted from Gaussian output.

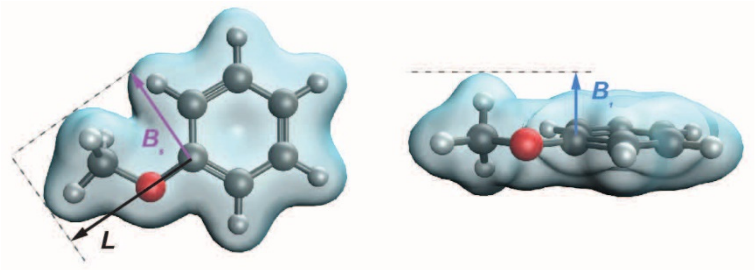

Figure S12. Sterimol parameters L, B_1 and B_5 illustration along the modified aromatic side-chain of *O*-Methyl-*L*-tyrosine **14**.

3. Multivariate linear regression model

The multivariate linear regression model was calculated using R (V 4.1.0)[8] and RStudio.[9] Graphic representations were produced using the package ggplot2.[10] Other packages used in code are: tidyr,[11] reshape2, [12] scales,[13] tibble,[14] caret,[15] plyr,[16] dplyr,[17] data.table,[18] knitr,[19] ggrepel[20] and extrafont.[21] IQmol[22] and CYLview[23] were used for molecular graphic representations.

The model development process evaluated all possible models within a given range of the number of variables. The maximum number of variables was set to be the number of samples divided by five, hence maximum of three variables where considered and the minimum was set to one variable. The resulting models were assessed for their statistical significance and then ranked by a leave-one-out cross-validation. A goodness-of-fit measure R^2 is provided together along with a leave-one-out cross-validation Q^2_{LOO}. The best model was chosen based on an internal validation in which the predicted values from all test sets are measured against the known results, and the Q^2 goodness-of-fit is measured by the models' ability to reproduce results. Leave-one-out cross validation is a private case of K-fold cross-validation in which the number of folds (K) is the number of samples in the data, such that each sample is treated once as the test set. The Q^2 acts as the validation's R^2 and a good value for Q^2 is a value that is close to the R^2. Thus, both measures are used for the model evaluation.

3.1. Model report

Formula	R.sq	Q.sq	MAE
output ~ Total + B1 + B5	0.8318340	0.7502347	2.941805

	Estimate	Std. Error	t value	Pr(>|t|)
(Intercept)	47.576000	0.8478527	56.113518	0.0000000
Total	-4.511473	0.9571308	-4.713539	0.0004052
B1	4.453420	0.9532281	4.671935	0.0004368
B5	7.018219	0.9491675	7.394078	0.0000052

$Q_{3\text{-fold}}$ cross validation

Q^2	MAE
0.7034725	3.499447

$Q_{5\text{-fold}}$ cross validation

Q^2	MAE
0.7286795	3.196038

Optimized geometry structures - xyz coordinates

This section contains xyz coordinates of the optimized structures of the aromatic ring side chain for each modification.

Tyrosine

14

C -4.4174428609 0.5830330484 1.6066868
C -4.1589906473 0.7906025496 2.9576710
C -3.3521577021 1.8496361381 3.3626763
C -2.8076829471 2.6966646869 2.4087663
C -3.061018577 2.4959108157 1.0597497
C -3.8678905646 1.4356716156 0.6650617
H -5.0476715119 -0.2471945017 1.3179687
N -4.7603998771 -0.1284347949 3.8572024
H -3.1481461916 2.0151090162 4.4136144
H -2.1806277385 3.5194018438 2.7266827
H -2.6332768151 3.1601693127 0.3212063
H -4.0713387911 1.270839407 -0.3849493
N -4.5345314332 0.0515023593 5.0512465
N -4.3855482325 0.1359946334 6.1556188

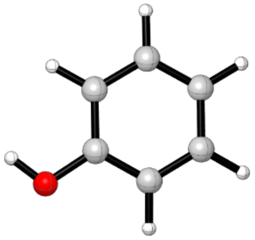

p-Acetyl-phenylalanine

17

C -3.1982493132 0.5801560998 1.9610102458
C -4.2748848912 0.8632111774 2.7971810026
C -4.8444359172 2.1350534947 2.774994215
C -4.3441042222 3.1089484744 1.9287678834
C -3.2685939827 2.8203294821 1.0959019902

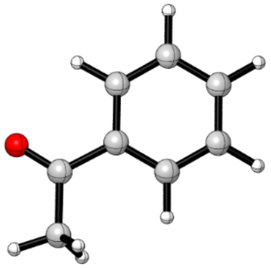

C -2.6962854551 1.5567810039 1.112229521

H -2.7450696509 -0.4025220067 1.9665613477

C -4.8515407097 -0.1586245684 3.7308460233

H -5.6802913391 2.3351256481 3.4323286679

H -4.7896853677 4.0950273331 1.9150903035

H -2.8771499586 3.5820756264 0.4339209866

H -1.8593575386 1.3311699466 0.4645517633

O -5.7855623311 0.1180825044 4.4435530951

C -4.2366922764 -1.5373995079 3.757027857

H -3.1818025301 -1.4799469043 4.0304786534

H -4.7712816979 -2.1446759023 4.4815902355

H -4.2966550984 -2.0011321713 2.7710253487

H-p-Phenyl-phenylalanine

22

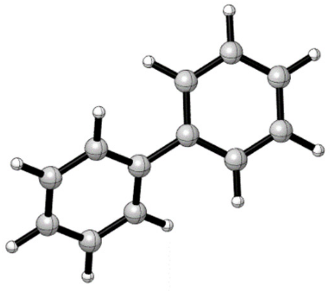

C -3.420267799 0.2673826218 1.7820293054

C -4.2290644236 0.7015576787 2.8325109789

C -4.4204965571 2.0730872631 3.0010612247

C -3.821775927 2.984063274 2.1442651523

C -3.0187958161 2.5396347502 1.1029556077

C -2.8200511508 1.1773990874 0.9253379729

H -3.2419587988 -0.7928882053 1.6521202557

C -4.8694264748 -0.2710328883 3.7482251802

H -5.0647273254 2.4255836465 3.7970175499

H	-3.989035794	4.0440822182	2.285725629
H	-2.5508840586	3.2502268826	0.4342569617
H	-2.1889132684	0.8218187577	0.1210604549
C	-4.9596094375	-0.0108330077	5.1158200373
C	-5.3967012105	-1.4680277651	3.263092426
H	-4.5338992207	0.9028255262	5.5118428854
C	-5.996904424	-2.3781314424	4.1197072164
C	-5.5585152585	-0.9207251499	5.9736394337
C	-6.0798623312	-2.1081056284	5.4787235481
H	-5.6114116512	-0.7051514811	7.0330517415
H	-6.4079596526	-3.2977413796	3.7234962601
H	-6.5478197758	-2.8183616344	6.1477180983
H	-5.3564286943	-1.674258484	2.20069261

p-Azido-phenylalanine

14

C	-4.4174428609	0.5830330484	1.6066868
C	-4.1589906473	0.7906025496	2.9576710
C	-3.3521577021	1.8496361381	3.3626763
C	-2.8076829471	2.6966646869	2.4087663
C	-3.061018577	2.4959108157	1.0597497
C	-3.8678905646	1.4356716156	0.6650617
H	-5.0476715119	-0.2471945017	1.3179687
N	-4.7603998771	-0.1284347949	3.8572024

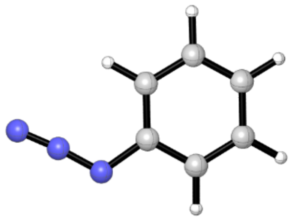

H -3.1481461916 2.0151090162 4.4136144

H -2.1806277385 3.5194018438 2.7266827

H -2.6332768151 3.1601693127 0.3212063

H -4.0713387911 1.270839407 -0.3849493

N -4.5345314332 0.0515023593 5.0512465

N -4.3855482325 0.1359946334 6.1556188

Azophenyl - trans

24

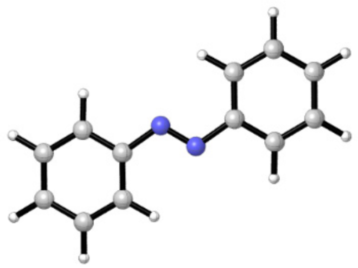

C -4.9208268568 1.0979925944 1.82702259

C -4.109791666 0.6633565754 2.8678697777

C -2.8149028654 1.1615893973 3.0076765625

C -2.3459563765 2.0931071135 2.0995359667

C -3.1567560954 2.5305871642 1.0553306964

C -4.4441763933 2.0326370578 0.9195842466

H -5.9203872773 0.6905547202 1.7481260156

N -4.6966251675 -0.2964214614 3.7412588054

H -2.1993554942 0.8110298273 3.8239031677

H -1.3419403525 2.4843569424 2.2015805236

H -2.7807864282 3.2599197745 0.3495825543

H -5.0760830695 2.370955859 0.1090873718

N -3.9693580839 -0.6767547177 4.6644865321

C -3.7449719611 -2.0695812307 6.5799220638

C -5.8505717349 -2.1352063446 5.3988906656

C -4.221280276 -3.0037680251 7.488003633

C -4.5560017701 -1.6361133124 5.5385686873

C -6.3191740819 -3.066285137 6.3076471774

H -6.466141943 -1.7856698094 4.5822375606

C -5.5083807024 -3.5025296633 7.352398626

H -3.5893706817 -3.3411517748 8.2989018973

H -7.3229372107 -3.4581491116 6.2055968147

H -5.8839829536 -4.2315338784 8.05868494

H -2.7456118283 -1.6616368896 6.6587682038

Azophenyl - cis

24

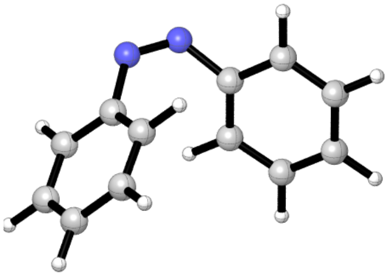

C 1.1888009959 -0.11327699 1.12460585

C 1.4766404796 0.76404120 0.08703659

C 2.6024699945 0.57244859 -0.70689437

C 3.418436196 -0.52378527 -0.47700846

C 3.1081587607 -1.43243981 0.52700825

C 1.9914851388 -1.22539529 1.32608869

H 0.3343322014 0.08693273 1.75835728

N 0.654444672 1.93697838 -0.01833210

H 2.8353998395 1.27952036 -1.49222747

H 4.2994654831 -0.67105159 -1.08792159

H 3.7445367641 -2.29094754 0.69587757

H 1.7551824625 -1.92164097 2.12008079

N	0.1712389474	2.31810572	-1.08442776
C	-0.1557227863	0.22580365	-2.36689782
C	0.639551928	2.23311882	-3.44452657
C	-0.1532359703	-0.42305345	-3.59140696
C	0.2592576993	1.55140849	-2.29573667
C	0.6709401949	1.56670142	-4.65966726
H	0.9105422609	3.27838591	-3.36824943
C	0.2737158197	0.23824134	-4.73609205
H	-0.4831767453	-1.45198396	-3.65108131
H	0.9885579271	2.09072713	-5.55159962
H	0.2813297739	-0.27639431	-5.68772949
H	-0.4804912877	-0.28655619	-1.47081208

P-Benzoyl-Phenylalanine

24

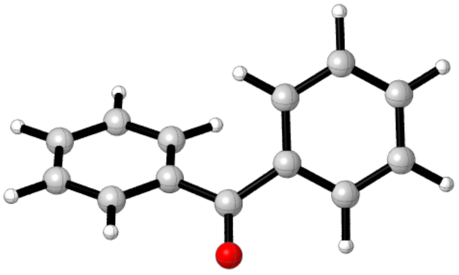

C	-5.846547934	0.9704655198	1.6952662
C	-6.2162400597	0.2819072893	2.8487948
C	-7.5649273163	0.1541892727	3.1716519
C	-8.5333924436	0.7059830603	2.3449696
C	-8.1597548242	1.3706006768	1.1855247
C	-6.814648491	1.5015863453	0.8602232
H	-4.7930881631	1.0804717041	1.4732671
C	-5.1277480629	-0.2307023387	3.7404454
H	-7.8568664446	-0.3564144404	4.0803906

H -9.5795200315 0.6181071845 2.6073009

H -8.9166999295 1.79226661 0.5367561

H -6.5236562161 2.0238498146 -0.0417676

O -4.0596058609 0.3353432726 3.7827316

C -5.3681474045 -1.4475516652 4.5795514

C -4.6632880135 -1.5665672025 5.7755573

C -6.3452010577 -3.6174589835 4.9532567

H -6.7377952952 -2.4046286364 3.2290202

C -4.8174609757 -2.6924237422 6.5662900

H -3.9966189075 -0.7649087219 6.0654871

C -5.6582944993 -3.7199607999 6.1546203

H -6.9869900245 -4.4241634649 4.6242370

H -4.2786004874 -2.7748373743 7.5010913

H -5.7732923802 -4.6037230405 6.7689499

C -6.2084257772 -2.4799440898 4.1702114

3-(2-Naphthyl)-alanine

18

C -2.9144631008 1.13838247 3.03494618

C -4.2680723476 0.77447163 2.82862240

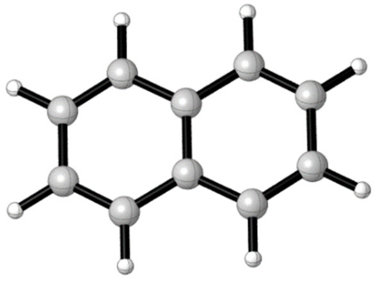

C -4.9665265723 1.32500087 1.72421411

C -4.2892025244 2.22163465 0.86162490

C -2.983667602 2.55417298 1.08556256

C -2.2876344133 2.00542645 2.18632018

H -2.3853873791 0.71487459 3.88027073

C -4.9454179899 -0.12219778 3.69124725

C -6.320068364 0.96125344 1.51772752

H -4.8278943963 2.63920116 0.01934002

H -2.4745593346 3.24012383 0.42098914

H -1.2533441779 2.27740307 2.35220280

H -4.4069295483 -0.53986136 4.53354610

C -6.2509813033 -0.45449626 3.46698608

H -7.9813153535 -0.17780990 2.20049337

C -6.9470705566 0.09415457 2.36634704

H -6.7600781218 -1.14036873 4.13147313

H -6.8489727242 1.38487121 0.67232888

O-Benzyl-tyrosine

26

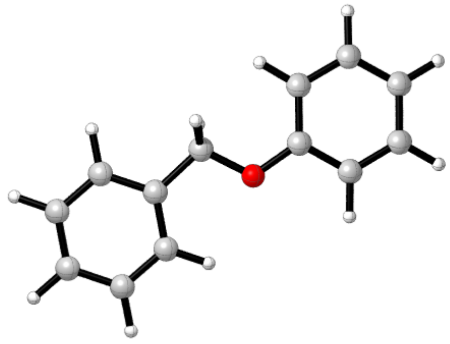

C -3.0386477985 0.8575837018 2.3502325131

C -4.4238418277 0.7931801971 2.498743742

C -5.2328528082 1.7411909017 1.8817785849

C -4.644959393 2.7470344327 1.1200143902

C -3.2717269794 2.81636479 0.9688309266

C -2.4714265669 1.8621802427 1.5909166069

H -2.4323029905 0.1077598365 2.8407236693

O -4.8892901334 -0.2290644068 3.2634039055

H -6.3075972129 1.7107375621 1.9832891208

H -5.2803683845 3.4818442956 0.6424240095

H -2.8253251482 3.6020133323 0.3747842199

H -1.3952636296 1.90290832 1.4821559945

C -6.2809855189 -0.3404183511 3.442338368

H -6.774759062 -0.4540108311 2.4698047224

C -6.5946916845 -1.5283943383 4.3116623629

C -5.6042573213 -2.3517730451 4.8282322454

C -7.9286927036 -1.8024468585 4.6021227174

C -5.9466917815 -3.4370747182 5.6265261774

H -4.568189936 -2.1413166012 4.6043792674

C -8.2694326612 -2.8837753222 5.3974367352

H -8.706569578 -1.1617599583 4.2006976221

C -7.2754514703 -3.7066345586 5.913378011

H -5.1672575055 -4.0737817339 6.0250252194

H -9.3100968082 -3.0858197694 5.6156059827

H -7.5384529756 -4.5523440219 6.5351397773

H -6.6717859405 0.5717778223 3.9089520783

4-Bromo-phenylalanine

12

C -3.76524971 0.3078064 1.5596998

C -4.2085140381 0.7352369 2.8013260

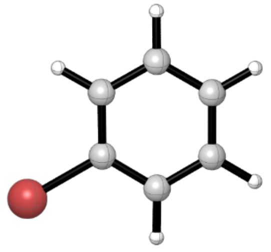

C -4.0675500916 2.0541781 3.2028020

C -3.4690676106 2.9603035 2.3383059

C -3.019258174 2.5500365 1.0912176

C -3.1687577302 1.2252037 0.7058699

H -3.8854645208 -0.7260163 1.2670774

Br -5.0247175711 -0.5103147 3.9749305

H -4.4206187106 2.3658269 4.1759863

H -3.3560094954 3.9917161 2.6458626

H -2.5535674614 3.2606492 0.4215523

H -2.8204731761 0.8978311 -0.2650624

4-Iodo-phenylalanine

12

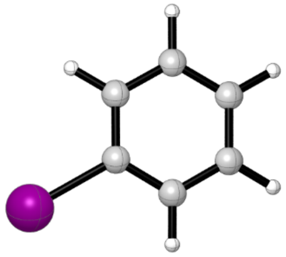

C -3.7654400253 0.3082711 1.5603483

C -4.2103995723 0.7323901 2.8039957

C -4.0675417096 2.0534729 3.2023601

C -3.468906691 2.9602931 2.3379363

C -3.0190861527 2.5502848 1.0909882

C -3.1686622716 1.2256005 0.7058759

H -3.8822627961 -0.7239836 1.2608577

I -5.1108331407 -0.6416902 4.0986782

H -4.4182107985 2.3724062 4.1740633

H -3.3559798198 3.9917375 2.6458258

H -2.5533435555 3.2609615 0.4212681

H -2.8204403569 0.8978827 -0.2650865

4-Chloro-phenylalanine

12

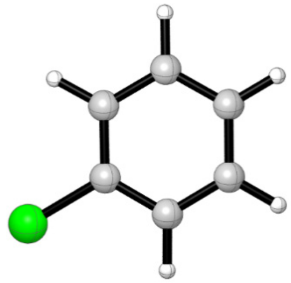

C	-3.7653394681	0.3081708448	1.5601268
C	-4.2089016281	0.734627814	2.8019470
C	-4.0675028395	2.0537128037	3.2024719
C	-3.4692694415	2.9599661436	2.3385518
C	-3.0192312329	2.5500951114	1.0911806
C	-3.1689749313	1.2248966143	0.7062062
H	-3.8875261385	-0.7264494786	1.2715475
Cl	-4.9557059057	-0.4050658087	3.8757365
H	-4.4219048406	2.3611416168	4.1763988
H	-3.3561024259	3.9912550652	2.6457735
H	-2.5535144238	3.2607101661	0.4214606
H	-2.820649804	0.8979469775	-0.2646308

O-Methyl-tyrosine

16

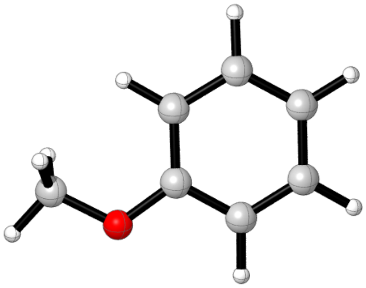

C	-3.2863051543	1.6898255322	3.1079578
C	-4.3312489862	0.8822287379	2.6589888
C	-4.8056009453	1.0214632446	1.3589642
C	-4.2282240377	1.9696602062	0.5195420
C	-3.1916066752	2.7735885171	0.9591620
C	-2.7242764601	2.6261266647	2.2620007
H	-2.9366302235	1.5607475125	4.1235590

O -4.8201071061 -0.0084674055 3.5586614

H -5.6136035007 0.4062859265 0.9908454

H -4.6023730888 2.0728445781 -0.4909975

H -2.7497543078 3.5069724495 0.2984696

H -1.913970583 3.2475129302 2.6211707

C -5.8779433433 -0.8430176018 3.1481980

H -6.7583118206 -0.2581588074 2.8672926

H -5.5808994995 -1.4742287161 2.3059880

H -6.1193560478 -1.4709247586 4.0017331

4-Methyl-phenylalanine

15

C -3.7743968906 0.3218771937 1.5600242

C -4.2350905479 0.7165336113 2.8126808

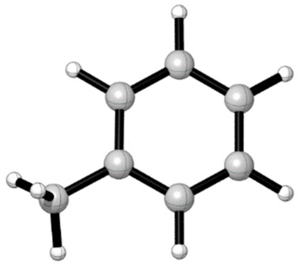

C -4.0738515495 2.0485009195 3.1864445

C -3.4653413213 2.9599296723 2.3360842

C -3.0072488224 2.5526476061 1.0905353

C -3.1647731178 1.2294063217 0.7047009

H -3.8975435283 -0.7095081388 1.2503391

C -4.8675519754 -0.2731714705 3.7521147

H -4.4328007754 2.3742990195 4.1560892

H -3.3516091263 3.9912186117 2.6449692

H -2.5347397753 3.2626095388 0.4242899

H -2.8154414867 0.9024116013 -0.2663445

H   -5.6676368267   0.1888617437   4.3306232

H   -5.2822957272   -1.1223350754   3.2098663

H   -4.1303218093   -0.658999305   4.4597827

p-Trifluoromethyl-phenylalanine

15

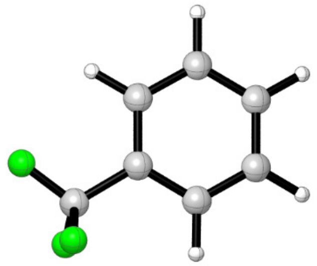

C   -3.1601445415   0.5919883973   2.0225041

C   -4.2487453097   0.8933086719   2.8323253

C   -4.8423224781   2.1441176998   2.7849999

C   -4.3396673158   3.1051275033   1.9172988

C   -3.2539823822   2.8111269567   1.1069538

C   -2.6636236754   1.553825886   1.1593297

H   -2.7074307665   -0.3902270835   2.0729921

C   -4.7713577351   -0.1756237236   3.7494857

H   -5.6879239666   2.3657279381   3.4210990

H   -4.7986670274   4.0839030921   1.8771792

H   -2.8643405762   3.5621361676   0.4322004

H   -1.8158982348   1.3247153273   0.5275681

F   -3.8075854382   -0.6567915027   4.5469240

F   -5.2542386623   -1.2214816438   3.0633011

F   -5.7549222601   0.2645307236   4.5391303

H-Tyr(propargyl)-OH

18

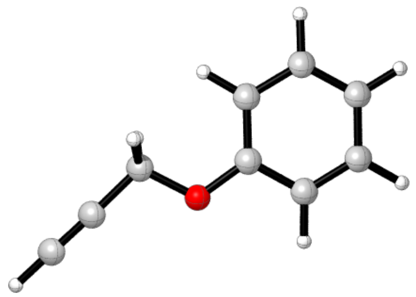

C	-3.3420938868	1.63232679	3.1301122
C	-4.3836980741	0.85350366	2.6272096
C	-4.8161013643	1.03057909	1.3178930
C	-4.197993509	1.99047774	0.5208241
C	-3.1643062394	2.76629608	1.0128366
C	-2.7401734965	2.57974726	2.3259064
H	-3.0275389423	1.47191495	4.1525990
O	-4.9112363685	-0.05176174	3.4960078
H	-5.6210155197	0.43969126	0.9057134
H	-4.5387616132	2.12403065	-0.4978448
H	-2.6910498049	3.50902695	0.3853508
H	-1.9324221508	3.17951209	2.7251173
C	-5.9691555731	-0.85768696	3.0238426
H	-5.6436611949	-1.44745536	2.1600202
C	-6.3957050885	-1.75435134	4.0929473
C	-6.7667439954	-2.50109893	4.9498850
H	-7.0918330952	-3.16221818	5.7171209
H	-6.8136392633	-0.23419201	2.7107184

O-tert-Butyl-tyrosine

25

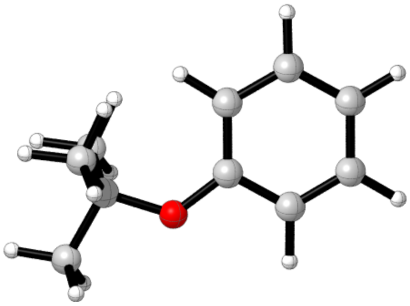

C	-3.3003480252	1.6943484401	3.104991356
C	-4.3244279209	0.9707433014	2.4865735182
C	-4.6436943485	1.2554598496	1.1611981522
C	-3.9435468288	2.2475238592	0.4806653362
C	-2.9307959093	2.9624920358	1.0935731023
C	-2.6141517634	2.6756765298	2.4174997975
H	-3.0658663514	1.459777334	4.1348032287
O	-4.9049829909	0.0411391552	3.2860770966
H	-5.4237516803	0.7256972038	0.6418794012
H	-4.2056986012	2.4549831304	-0.5491145648
H	-2.394979138	3.7305668921	0.5529261873
H	-1.8252475713	3.2219401819	2.9186761191
C	-5.9824041913	-0.8303360959	2.9040066916
C	-5.5597172835	-1.7820548597	1.7877387609
C	-6.2220911334	-1.6342711454	4.1775695416
H	-7.4636117665	0.6584241675	3.3769594052
H	-7.1715207753	0.5206243222	1.6418942593
H	-8.0850250299	-0.7318162574	2.4765686358
H	-4.6277302978	-2.2767576385	2.0632024656
H	-6.3286008737	-2.5448164851	1.6571259331
H	-5.4136034713	-1.2878564985	0.8307606017

H -7.0292332989 -2.3513003828 4.0272906433

H -5.3163294881 -2.1738924137 4.4539356712

H -6.4902992513 -0.9659297194 4.9957244838

C -7.24568116 -0.0412333367 2.5693558963

References

1. Frisch, M. J.; Trucks, G. W.; Schlegel, H. B.; Scuseria, G. E.; Robb, M. A.; Cheeseman, J. R.; Scalmani, G.; Barone, V.; Petersson, G. A.; Nakatsuji, H.; Li, X.; Caricato, M.; Marenich, A. V.; Bloino, J.; Janesko, B. G.; Gomperts, R.; Mennucci, B.; Hratchian, H. P.; Ortiz, J. V.; Izmaylov, A. F.; Sonnenberg, J. L.; Williams-Young, D.; Ding, F.; Lipparini, F.; Egidi, F.; Goings, J.; Peng, B.; Petrone, A.; Henderson, T.; Ranasinghe, D.; Zakrzewski, V. G.; Gao, J.; Rega, N.; Zheng, G.; Liang, W.; Hada, M.; Ehara, M.; Toyota, K.; Fukuda, R.; Hasegawa, J.; Ishida, M.; Nakajima, T.; Honda, Y.; Kitao, O.; Nakai, H.; Vreven, T.; Throssell, K.; Montgomery, J. A., Jr.; Peralta, J. E.; Ogliaro, F.; Bearpark, M. J.; Heyd, J. J.; Brothers, E. N.; Kudin, K. N.; Staroverov, V. N.; Keith, T. A.; Kobayashi, R.; Normand, J.; Raghavachari, K.; Rendell, A. P.; Burant, J. C.; Iyengar, S. S.; Tomasi, J.; Cossi, M.; Millam, J. M.; Klene, M.; Adamo, C.; Cammi, R.; Ochterski, J. W.; Martin, R. L.; Morokuma, K.; Farkas, O.; Foresman, J. B.; Fox, D. J. Gaussian 16, Revision C.01, Gaussian, Inc.: Wallingford CT, **2016**.

2. Zhao, Y.; Truhlar, D. G. The M06 Suite of Density Functionals for Main Group Thermochemistry, Thermochemical Kinetics, Noncovalent Interactions, Excited States, and Transition Elements: Two New Functionals and Systematic Testing of Four M06-Class Functionals and 12 Other Functionals. Theoretical Chemistry Accounts **2007**, 120 (1-3), 215– 241. https://doi.org/10.1007/s00214-007-0310-x

3. Valero, R.; Gomes, J. R. B.; Truhlar, D. G.; Illas, F. Good Performance of the M06 Family of Hybrid Meta Generalized Gradient Approximation Density Functionals on a Difficult Case: CO Adsorption on MgO(001). The Journal of Chemical Physics **2008**, 129 (12), 124710. https://doi.org/10.1063/1.2982923.

4. Weigend, F.; Ahlrichs, R. Balanced Basis Sets of Split Valence, Triple Zeta Valence and Quadruple Zeta Valence Quality for H to Rn: Design and Assessment of Accuracy. Physical Chemistry Chemical Physics **2005**, 7 (18), 3297. https://doi.org/10.1039/b508541a.

5. Weigend, F. Accurate Coulomb-Fitting Basis Sets for H to Rn. Physical Chemistry Chemical Physics **2006**, 8 (9), 1057. https://doi.org/10.1039/b515623h.

6. Merrick, J. P.; Moran, D.; Radom, L. An Evaluation of Harmonic Vibrational Frequency Scale Factors. The Journal of Physical Chemistry A **2007**, 111 (45), 11683–11700. https://doi.org/10.1021/jp073974n.

7. Verloop, A.; Tipker, J. Use of Linear Free Energy Related and Other Parameters in the Study of Fungicidal Selectivity. Pesticide Science **1976**, 7 (4), 379–390. https://doi.org/10.1002/ps.2780070410.

8. R Core Team. R: A language and environment for statistical computing. R Foundation for Statistical Computing: Vienna, Austria, **2014**. https://www.r-project.org/.

9. RStudio Team. RStudio: Integrated Development for R. RStudio, PBC.: Boston, MA, **2020**. http://www.rstudio.com/.

10. Wickham, H. ggplot2: Elegant Graphics for Data Analysis. Springer-Verlag: New York, **2016**. https://ggplot2.tidyverse.org.

11. Wickham, H. and Henry, L. tidyr: Tidy Messy Data. R package version 1.1.0.: **2019**. https://CRAN.R-project.org/package=tidyr.

12. Wickham, H. (2007). Reshaping Data with the reshape Package. Journal of Statistical Software, **2007**, 21 (12), 1-20. http://www.jstatsoft.org/v21/i12/.

13. Wickham, H. and Seidel, D. scales: Scale Functions for Visualization. R package version 1.1.1.: **2020**. https://CRAN.R-project.org/package=scales.

14. Muller, K. and Wickham, H. tibble: Simple Data Frames. R package version 3.0.3.: **2020**. https://CRAN.R-project.org/package=tibble.

15. Kuhn, M. caret: Classification and Regression Training. R package version 6.0-86.: **2020**. https://CRAN.R-project.org/package=caret

16. Wickham, H. The Split-Apply-Combine Strategy for Data Analysis. Journal of Statistical Software, **2011**, 40 (1), 1-29. http://www.jstatsoft.org/v40/i01/.

17. Wickham, H.; Fransois, R.; Henry, L.; Muller, K. dplyr: A Grammar of Data Manipulation. R package version 1.0.0.: 2020. https://CRAN.R-project.org/package=dplyr.

18. Dowle, M. and Srinivasan, A. data.table: Extension of data.frame. R package version 1.12.8.: **2019**. https://CRAN.R-project.org/package=data.table.

19. Domencich, T. and McFadden, D. L. Urban Travel Demand: A Behavioral Analysis. NorthHolland Publishing Co.: Netherlands, **1975**.

20. Slowikowski, K. ggrepel: Automatically Position Non-Overlapping Text Labels with 'ggplot2'. R package version 0.9.1.:2021. https://CRAN.R-project.org/package=ggrepel.

21. Chang, W. extrafont: Tools for using fonts. R package version 0.17.: **2014**. https://cran.r-project.org/web/packages/extrafont/index.html.

22. Gilbert, A. iQmol. Version 2.15; Australian National University: **2015**. http://iqmol.org.

23. Legault, C. Y. CYLview. Version 1.0b; Université de Sherbrooke: **2009**. http://www.cylview.org.
